# Cytokine enrichment in deep cerebellar nuclei is contributed by multiple glial populations and linked to reduced amyloid plaque pathology

**DOI:** 10.1186/s12974-023-02913-8

**Published:** 2023-11-17

**Authors:** Jessica R. Gaunt, Norliyana Zainolabidin, Alaric K. K. Yip, Jia Min Tan, Aloysius Y. T. Low, Albert I. Chen, Toh Hean Ch’ng

**Affiliations:** 1 Lee Kong Chian School of Medicine, Nanyang Technological University, Clinical Science Building, 11 Mandalay Road, Singapore, 308232 Singapore; 2 School of Biological Science, Nanyang Technological University, Singapore, 63755 Singapore; 3 Department of Biology, University of Pennsylvania, Philadelphia, PA USA; 4 Center for Aging Research, Scintillon Institute, 6868 Nancy Ridge Drive, San Diego, CA 92121 USA; 5 Molecular Neurobiology Laboratory, Salk Institute, La Jolla, CA 92037 USA

**Keywords:** Alzheimer’s disease, Microglia, Interferon, Deep cerebellar nuclei, CSF1R inhibitor, Entorhinal cortex

## Abstract

Alzheimer’s disease (AD) pathology and amyloid-beta (Aβ) plaque deposition progress slowly in the cerebellum compared to other brain regions, while the entorhinal cortex (EC) is one of the most vulnerable regions. Using a knock-in AD mouse model (*App* KI), we show that within the cerebellum, the deep cerebellar nuclei (DCN) has particularly low accumulation of Aβ plaques. To identify factors that might underlie differences in the progression of AD-associated neuropathology across regions, we profiled gene expression in single nuclei (snRNAseq) across all cell types in the DCN and EC of wild-type (WT) and *App* KI male mice at age 7 months. We found differences in expression of genes associated with inflammatory activation, PI3K–AKT signalling, and neuron support functions between both regions and genotypes. In WT mice, the expression of interferon-response genes in microglia is higher in the DCN than the EC and this enrichment is confirmed by RNA in situ hybridisation, and measurement of inflammatory cytokines by protein array. Our analyses also revealed that multiple glial populations are responsible for establishing this cytokine-enriched niche. Furthermore, homogenates derived from the DCN induced inflammatory gene expression in BV2 microglia. We also assessed the relationship between the DCN microenvironment and Aβ pathology by depleting microglia using a CSF1R inhibitor PLX5622 and saw that, surprisingly, the expression of a subset of inflammatory cytokines was increased while plaque abundance in the DCN was further reduced. Overall, our study revealed the presence of a cytokine-enriched microenvironment unique to the DCN that when modulated, can alter plaque deposition.

**Supplementary Information:**

The online version contains supplementary material available at 10.1186/s12974-023-02913-8.

## Introduction

Alzheimer’s disease (AD) is a progressive neurodegenerative disorder that accounts for 60–80% of total dementia cases [1]. The clinical presentation of AD is paralleled by the progression of pathology across brain regions with different functional specialisations [2, 3]. The hippocampus and entorhinal cortex (EC), which are critical for the encoding of memories of events, places and time, are the first to show neuropathological hallmarks of AD, while the cerebellum, which is primarily involved in motor function, is spared until much later [3–6]. A similar progression of pathology is also observed in various mouse models of Alzheimer’s disease including, but not limited to, APP/PS1 [7], 5xFAD [8] and APPswe/PSEn1dE9 [9]. Overall, the differences in pathology between the cerebellum and EC are well conserved between human patients and animal models of the disease.

The major features of AD pathology are neurotoxic protein accumulations in the form of Aβ plaques and neurofibrillary tangles [6]. Aβ plaques are aggregates of Aβ protein fragments, formed by cleavage of amyloid precursor protein (APP) at specific sites [10]. Histopathological studies of AD brains have shown that there are fewer Aβ plaques in the cerebellum, with a delay in the appearance of pathology relative to the EC and hippocampus [5, 6]. With more sensitive histological techniques, regional differences in Aβ pathologies within the cerebellum have been reported, with the majority of Aβ deposits being found in the molecular, granular and Purkinje cell layers of the cerebellar cortex, but still largely absent in the deep cerebellar nuclei [5, 11–13]. Importantly, tau neurofibrillary tangles have never been widely detected in the cerebellum [5, 14]. Because of the low levels of Aβ pathology, the cerebellum is routinely employed as a reference region to calculate cortical-to-cerebellum standardised uptake value ratio (SUVr) in PET imaging studies to quantify amyloid load [15–17].

By studying how Aβ pathology develops differently in the cerebellum in comparison to the regions affected earliest in the disease, and why specific regions of the cerebellum such as the DCN are relatively devoid of pathology, we can gain insight into the mechanisms of AD and potentially identify protective mechanisms that may lead to the development of novel treatments. We reasoned that the differences in pathologies may arise due to the unique microenvironments established, in part, by the distinct cell type compositions of the two brain regions. To identify differentially regulated genes and cellular pathways in these regions that may influence Aβ pathology, we profiled single-nuclei transcriptomes of EC and DCN in both wild-type (WT) and AD model mice. For this study, we examined the homozygous *App*^*NL-G-F/NL-G-F*^ knock-in (*App* KI) mouse which expresses a humanised mutant form of APP containing three mutations associated with familial Alzheimer’s disease [18]. In these mice, mutations in the Aβ region of APP promote cortical deposition of plaques in mice beginning as early as 2 months and reaching saturation by 7 months [18]. Importantly, the expression of APP is regulated by the endogenous mouse promoter which avoids artefacts of overexpression. In addition, no increases in hyperphosphorylated tau or neurofibrillary tangles have been reported in *App* KI animals [18, 19].

Here, we report that the DCN exhibits an elevated cytokine expression that is independent of Aβ pathology. While the cerebellum has been previously reported to be enriched with a population of interferon-response-associated microglia, our data extend this observation by showing that multiple cell types in the DCN exhibit a basally elevated cytokine transcriptional signature, indicating a coordinated cellular program to establish the immune niche. We confirmed that the cytokine enrichment extends to the protein level by performing immunoassays as well as treatment of BV2 microglia cell lines with brain tissue homogenates to show a robust inflammatory transcriptional response. Finally, we depleted microglia in *App* KI brains and show altered plaque deposition in the DCN that is consistent with the hypothesis that the cytokine-enriched microenvironment plays a critical role in Aβ plaque deposition.

## Results

### Distinctions in Alzheimer’s disease pathology between EC and DCN

**Fig. 1:**
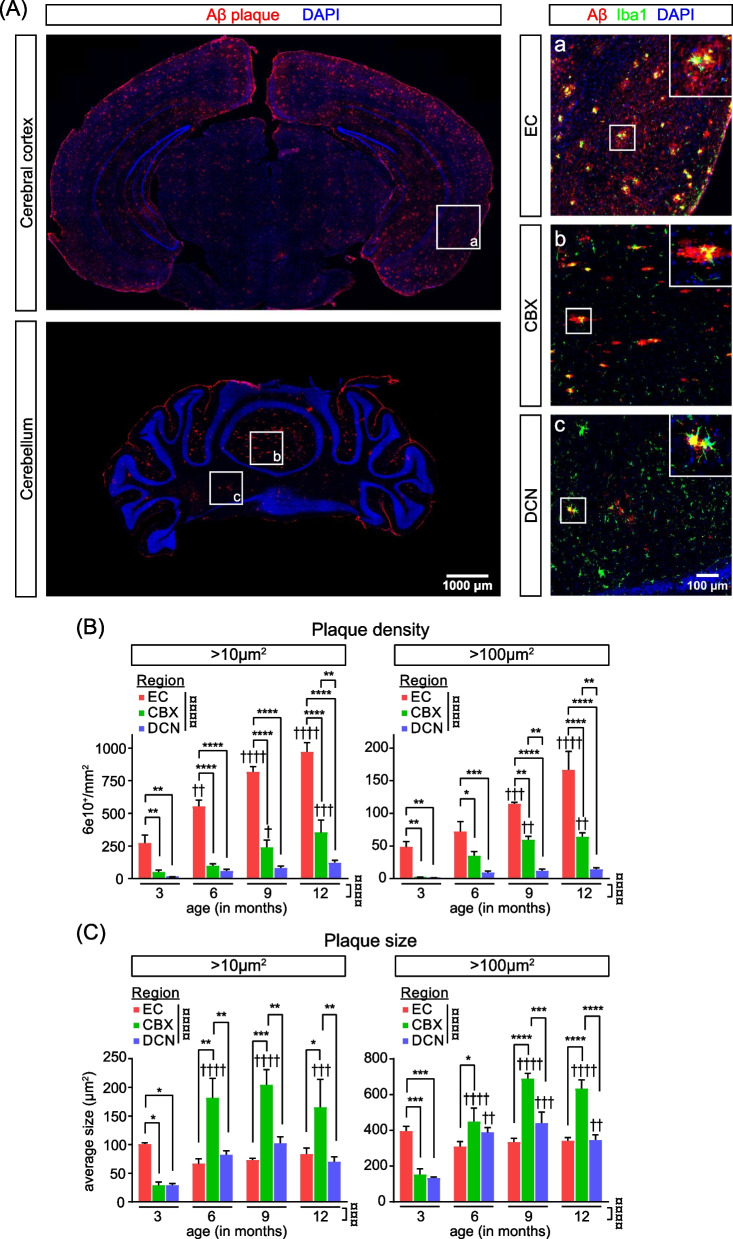
Amyloid-beta plaque burden in EC and DCN of *App KI* mice. **A** Immunohistochemistry of brain sections from 6-month-old *App*^*NL-G-F*^ mice using 6E10 antibody to detect Aβ plaques (red), IBA1 to detect microglia (green), and DAPI to stain nuclei (blue). Images were taken at 20X magnification. Left: regions of interest (ROIs) are indicated on whole brain sections: entorhinal cortex (EC, a), cerebellar cortex (CBX, b) and deep cerebellar nuclei (DCN, c). Scale bar at 1000 µm. Right: ROIs with an example of an Aβ plaque in each region indicated and enlarged in the top-right corner. Scale bar at 100 µm. **B** Density of 6E10^+^ positive plaques per mm^2^ in the EC, CBX and DCN using size filters of > 10 μm^2^ and > 100 μm^2^. Quantification was performed on *N* = 3 subjects, *n* = 3 sections/subject. Graphs indicate mean ± SEM. **C** Quantification of the average size of 6E10^+^ plaques per mm^2^ using size filters of > 10 μm^2^ and > 100 μm^2^. Graphs indicate mean ± SEM. Two-way ANOVAs were performed on within-subject means to test for main effects of region and genotype and interaction effects, followed by Tukey HSD post hoc tests indicated by currency sign (¤). Asterisks (*) indicate within-timepoint differences between regions. Daggers (†) above bar indicate between-timepoint differences within regions. * p < 0.05, ** p < 0.01, *** p < 0.001, **** p < *0.0001*

In the EC of *App* KI mice, Aβ plaques, assessed by immunohistochemistry using 6E10 antibody, are already present at 3 months of age and plaque load increases steeply over the following months (Fig. 1A, B). In contrast, the cerebellum is virtually devoid of Aβ plaques at 3 months and deposition increases steadily over time, with the overall density at 12 months still significantly lower than in the EC (Fig. 1A, B). Within the cerebellum, plaque abundance in the DCN is strikingly low and increases very gradually over time (Fig. 1A, B). Surprisingly, the average plaque size measured in the EC does not appear to change much over time, while plaques in the cerebellar cortex continue to increase in size before reaching a plateau at 9 months of age (Fig. 1A and C). It is notable that even though the density of plaques is low in the cerebellar cortex, on average plaques are larger relative to both the EC and DCN (Fig. 1A and 1C). Furthermore, plaques in the vermis of the cerebellar cortex also have a distinctly elongated morphology that is consistent with observations in both humans and APP/PS1 transgenic mice (Fig. 1A) [5, 20, 21]. Overall, we observed significantly fewer Aβ plaques in the cerebellum, and in particular the DCN in *App* KI mice.

### Analysis of single-nuclei transcriptomes from EC and DCN in *App* KI mice

We next used snRNAseq to characterise individual transcriptomes for all cell types in the DCN and EC and analyse expression differences associated with *App* KI pathology. We assessed tissues from 7-month-old male mice, as Aβ pathology at this time point is substantial in EC but only beginning to emerge in the cerebellum, with particularly few extracellular Aβ deposits in the DCN. We dissected the EC and DCN (Additional file 1: Fig. S1; *N* = 2 WT and *N* = 2 *App* KI for a total of 8 samples), then isolated nuclei in parallel using a sucrose cushion ultracentrifugation method and performed 10X droplet-based sequencing (Additional file 1: Fig. S2) [22]. To enhance read depth and improve cell type characterisation, we aligned reads to a pre-mRNA reference genome as a relatively high proportion of nuclear RNA reads originate from intronic regions [23].

**Fig. 2:**
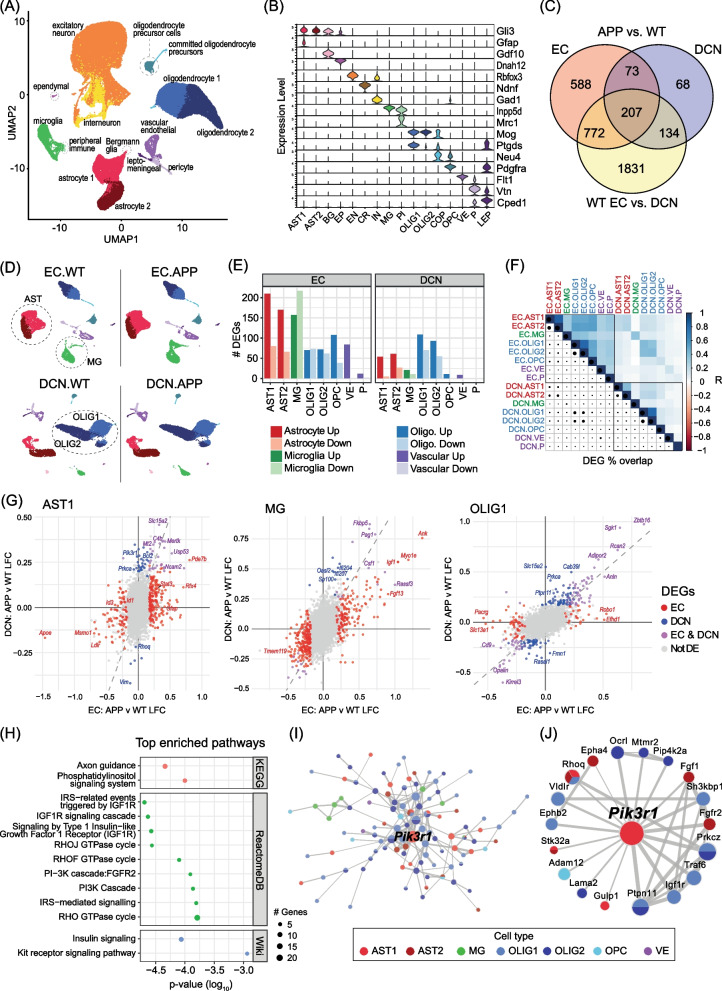
DEG analyses of snRNAseq data indicate shared and distinct pathways regulated in EC and DCN of *App* KI mice. **A** Integrated UMAP of single-nucleus transcriptomes for all conditions showing main cell types. **B** Violin plots showing canonical marker gene expression for each labelled cell type: astrocyte 1 [AST1], astrocyte 2 [AST2], Bergmann glia [BG], ependymal [EP], excitatory neurons [EN], Cajal Retzius [CR], interneuron [IN], microglia [MG], peripheral immune [PI], oligodendrocyte 1 [OLIG1], oligodendrocyte 2 [OLIG2], committed oligodendrocyte precursor cells [COP], oligodendrocyte precursor cells [OPC], vascular endothelial [VE], pericytes [P] and leptomeningeal cells [LEP]. **C** Venn diagram showing overlap in differentially expressed genes (DEGs) found in glial and vascular cells, regardless of cell type, for following DE tests: APP vs WT in EC, APP vs WT in DCN, and EC vs DCN in WT mice. **D** UMAPs for glia and vascular cells in EC and DCN separated by genotype. Data are integrated separately for each region. Dotted regions highlight cell types with highest numbers of DEGs as indicated in (**E**): green: microglia (MG); red: astrocytes (AST); blue: oligodendrocytes (OLIG). **E** DEGs for each glial subtype in each region. “Up” and “Down” denote upregulated and downregulated DEGs in APP compared to WT, respectively. **F** Correlogram of APP vs. WT LFCs between regions. Upper-right triangle indicates strength of correlation (R) and lower-left triangle indicates % of shared/total DEGs. Darker blue squares and larger circles correspond to cell types that have more similar *App* KI pathology between regions. **G** LFC plots comparing differences in gene expression between genotypes in DCN vs. EC for astrocytes, microglia and oligodendrocyte lineage cell subtypes. DEGs in each region are indicated by colour (red: EC; blue: DCN; purple: EC and DCN; grey: not DE). **H** Top enriched pathways from KEGG, ReactomeDB and Wikipathways for *App* KI DCN-specific DEGs combined across all glia and vascular cells (228 genes). **I** STRING protein–protein interactions among *App* KI DCN-specific DEGs with the top hub gene *Pik3r1* labelled. Node size indicates node degree, node colour indicates cell type(s) and line thickness denotes strength of interaction. **J** *Pik3r1* protein–protein interactions (as in I) labelled and enlarged

Following pre-processing of each sample, we integrated the data and performed clustering, UMAP visualisation and identification of cell populations by expression of canonical markers (Fig. 2A, B). After filtering, a total of 83,332 individual transcriptomes were used in the final analysis (average 20,833 cells per condition). We identified all major cell types in both regions, including astrocytes, excitatory and inhibitory neurons, microglia, oligodendrocytes, oligodendrocyte precursor cells (OPCs), and vascular cells (endothelial, pericytes and leptomeningeal cells), as well as small numbers of ependymal cells and Bergmann glia in the DCN samples, Cajal Retzius cells in the EC, and committed oligodendrocyte precursors and peripheral immune cells in both regions, indicating that our method of nuclear isolation was robust across different cell types (Fig. 2A, B and Additional file 1: Fig. S3). Astrocytes and oligodendrocytes each formed two large subclusters, broadly corresponding to newly formed/myelinating (OLIG1) and mature (OLIG2) subtypes for oligodendrocytes, and fibrous (AST1) and protoplasmic (AST2) subtypes for astrocytes based on expression of marker genes [Fig. 2A, B, [24]]. Overall, we found excitatory neurons to be the most abundant cell type across all samples, accounting for 45.7% of our dataset. For glial subtypes, astrocytes, OPCs and microglia are more abundant in our EC samples, while oligodendrocytes are enriched in DCN samples (Additional file 1: Fig. S4). While utmost care was taken to isolate EC and DCN, some of the differences in the distribution of cell types profiled could be due to isolation of surrounding tissues.

### Shared and distinct molecular signatures of App KI pathology in the EC and DCN

To begin, we analysed differential expression between *App* KI and WT tissues in each region. For these and further downstream analyses, we chose to focus on glia as initial analysis of neuronal subtypes revealed diverse subpopulations that are highly divergent between regions, making comparative analyses more challenging to interpret. Moreover, there is now overwhelming evidence that glial subtypes play major roles in the pathophysiology of AD, with multiple risk genes for sporadic AD primarily expressed in glial cells [25–28].

Overall, we found more than three times as many differentially expressed genes (DEGs) in EC glia compared to DCN glia, of which 280 genes overlapped (Fig. 2C). Notably, more than half of *App* KI DEGs are also differentially expressed between regions in WT mice, suggesting that underlying differences in glial transcriptomes directly contribute to differences in resistance and vulnerability to pathology between regions (Fig. 2C). In the EC, robust differential expression between WT and *App* KI genotypes was found in all glial cells, with the greatest number of DEGs in microglia (374 genes), followed by astrocytes (290 and 236 genes in AST1 and AST2, respectively; Fig. 2D–F and Additional file 1: Fig. S5). UMAPs also show that the most striking differences in cellular state between genotypes are in microglia, with substantial transcriptional changes particularly evident in the EC (Fig. 2D). Several publications have already identified a subpopulation of microglia that emerges with the progression of AD pathology in mouse models identified as DAMs, ARMs, MGnD, etc. [29–33]. In accordance with these publications, we observed a significant upregulation of genes that define the AD-associated microglia including subsets involved in lipid metabolism and chemotaxis (e.g. *Fgf13, Myo1e, Igf1, Ccl3, Ctnna3, Ank*), with a corresponding downregulation of homeostatic genes (e.g. *Tmem119, P2ry12* and *Cx3cr1*; Fig. 2G and Additional file 1: Fig. S5). Like microglia, both subclusters of astrocytes, AST1 and AST2, exhibit a substantial number of DEGs, including those previously identified in ‘disease associated astrocytes’ (DAAs) in the hippocampus and cortex of 5xFAD mice (e.g. downregulation of *Id1*, *Id3* in AST1, and enrichment of *Gfap, Stat3, Mt2, C4b, Ncam2* and *Usp53* in both clusters; Fig. 2G and Additional file 1: Fig. S5) [34].

In the DCN, oligodendrocytes have the highest numbers of DEGs among glial populations in *App KI* mice (179 genes in OLIG1 and 148 genes in OLIG2; Fig. 2C–E). Expression fold changes in oligodendrocytes are also the most strongly correlated between regions (*r* = 0.407–0.434), with 25–30% of DEGs in the DCN shared with the EC for both oligodendrocyte subtypes (Fig. 2F). In both regions, the most robust DEGs in *App* KI oligodendrocytes include *Zbtb16, Sgk1, Rcan2, Adipor2, Snca, Anln* (upregulated) and *Cdk8* and *Kirrel3* (downregulated; Fig. 2G and Additional file 1: Fig. S5). All of these genes have been identified as risk factors or shown to have altered expression in AD [35–42]. For the microglia and astrocyte subpopulations in the DCN, we identified fewer DEGs than in the EC, likely reflective of the lower levels of Aβ pathology in the region (Fig. 2E–G). Only a small subset of these DEGs is shared with EC and linked to disease-associated microglia (DAMs) [29] and astrocytes (DAAs) [34].

While many of the EC-specific and shared DEGs are likely altered in response to Aβ plaques, changes in gene expression in the DCN are largely independent of plaque deposition and could instead be linked to pathways underlying resistance to Aβ pathology in the region, such as alternate amyloid clearance mechanisms. To explore common pathways modulated by *App* KI among glial populations in the DCN but not EC, we performed network analyses on DCN-specific DEGs combined across all glial subpopulations (228 genes). Enriched pathways across multiple databases (KEGG, ReactomeDB, Wikipathways) were linked to insulin receptor signalling, PI3K signalling cascade and Rho-GTPases (Fig. 2H). While each of these pathways are individually associated with diverse cellular functions, they are also remarkably interconnected. Activation of insulin receptors, along with Rho-GTPases, can trigger the PI3K–AKT signalling pathway, initiating cellular programs responsible for regulating metabolism, cell survival and cell growth [43–45]. Analysis of protein–protein interactions via STRING revealed additional genes linked to PI3K–AKT/Rho GTPase signalling, with the majority expressed in astrocytes (e.g. *Pik3r1, Fgf1, Prkca, Bcl2, Gulp1)* and oligodendrocytes (e.g. *Prkcz, Igf1r, Ptpn11, Pip4k2a, Rhoj*) which is to be expected as the bulk of DEGs are located in these glial cell types. In addition, we also identified a small cluster of interferon pathway genes expressed in microglia (*Ifi204, Ifi207*, *Oasl2, Sp100)* and oligodendrocyte lineage cells *(Ifi27, Aim2, Traf6*; Fig. 2I and J). The most highly connected node in the network is *Pik3r1* (Phosphoinositide-3-Kinase Regulatory Subunit 1; degree = 18), a major astrocytic hub gene and a subunit of PI3K that has been identified as a novel genetic variant in the progression of AD [46]. While the implications of the enrichment of insulin and PI3K–AKT/Rho GTPase signalling in the DCN remain to be determined, both pathways have been linked to AD [47–49].

### Microglia in the DCN are enriched for expression of genes associated with interferon-response and cytokine production

In addition to investigating differences in molecular pathways across genotypes and regions by DEG analysis, another approach is to identify modules of co-expressed genes defining cell subpopulations within each cell type using Monocle3 [50, 51]. These analyses are particularly suited for our dataset as they enable simultaneous identification of subsets of genes altered between regions and genotypes and are complementary to our DEG analyses.

We first analysed microglia as they showed the strongest transcriptional differences between *App* KI and WT mice in the EC. Numerous studies have found microglia to be affected early in the disease course of AD both in humans and in mouse models, with microglial states extensively profiled in RNAseq of sorted cells and in single cell transcriptomes [29–32]. Moreover, analysis of DEGs indicates a differential response to pathology between regions, with DCN-specific microglial DEGs, being primarily associated with interferon response pathways. While previous reports have shown increased presence of interferon-responsive microglia in the cortex of aging *App* KI animals, this population represents a small fraction of microglia in AD and has not been characterised in the DCN [30].

**Fig. 3:**
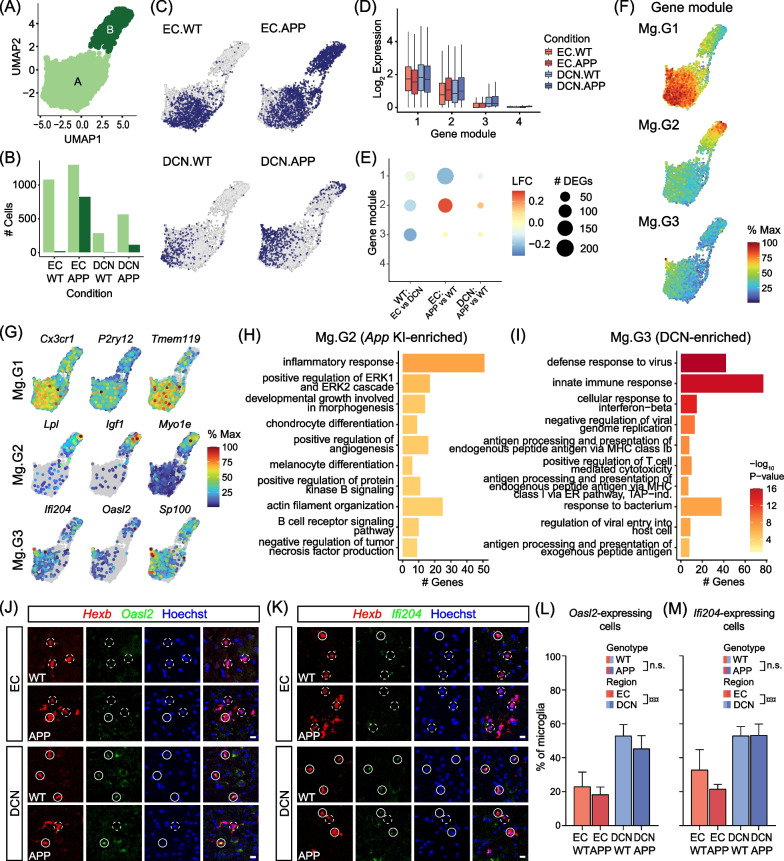
Subclustering of snRNAseq data reveals region- and genotype-specific subpopulations of microglia. **A** Subcluster UMAP of microglia nuclei transcriptomes showing two main clusters denoted as **A** and **B**. **B** Number of microglia per condition in each cluster. **C** Microglia UMAPs indicating cells from each condition (dark blue). **D** Boxplots of distributions of log_2_ gene expression for each module of co-expressed genes across conditions. Standard boxplot indicates box as median with upper and lower quartiles and whiskers as range excluding outliers (beyond 1.5*interquartile range from the upper/lower quartiles). **E** Dot plot indicating number of DEGs in each gene module (dot size) and average LFC (colour scale) for three DE tests: WT: EC vs. DCN, EC: APP vs. WT, DCN: APP vs. WT. **F** UMAPs showing aggregated expression of genes in the top 3 largest gene modules. Colour scale indicates expression in each cell relative to the cell with the highest expression of that module. **G** UMAPs of expression of selected top marker genes from each module showing percentage of maximum expression. **H**, **I** Gene Ontology analysis of biological processes enriched in genes in Mg.G2 (*App* KI-enriched; **H**) and Mg.G3 (DCN-enriched; **I**) compared to all detected microglial genes. Bar plot colour scale indicates -log_10_ p-value. **J** FISH assay to determine co-expression of IFN-regulated gene and microglia marker, *Oasl2* (green) and *Hexb* (red) respectively, in the EC (top panel) and DCN (bottom panel) of WT and *App* KI tissues. White closed circles indicate *Hexb*-positive microglia expressing *Oasl2* while broken circles indicate *Hexb*-positive cells that lack *Oasl2* expression. Scale bar at 10 µm. **K** As (**J**), but for the colocalisation of *Ifi204* and *Hexb* in the EC and DCN of WT and *App* KI tissue. Closed circles indicate *Hexb*-positive microglia that express *Ifi204* while broken circles indicate microglia that do not express *Ifi204*. Scale bar at 10 µm. (L-M) Percentage of *Hexb*-positive cells that express *Oasl2* (*N* = 3 subjects, *n* = 3 sections/subject; L) and *Ifi204* (*N* = 3 subjects, *n* = 3–4 sections/subject; M). Graphs indicate mean ± SEM. Two-way ANOVAs were performed on within-subject means to test for main effects of region and genotype and interaction effects, followed by Tukey HSD post hoc tests (¤). ** p < *0.01*

Cluster analysis of microglia with Monocle3 (4167 cells) detected two groups of microglia. The clusters were labelled Clusters A (3215 cells) and B (952 cells) and broadly correspond to homeostatic and disease-associated (DAM) microglial states, respectively (Fig. 3A–C). Separately, we then took genes that were differentially expressed across the microglia population and subdivided the genes into modules based on spatially correlated expression profiles (minimum 50 genes; Mg.G1-G4; Fig. 3D–F). The gene modules and cell clusters were overlaid on a UMAP to profile gene expression patterns defining the diversity of microglia within and across genotypes and brain regions. It is important to note that UMAPs of the average expression profiles of gene modules may partially correspond to cell clusters but are not equivalent.

The larger microglial cluster A contains cells from all conditions and has high expression of gene module Mg.G1 (559 genes; Figs. 3A, B and F). Mg.G1 genes include markers of homeostatic microglial states (e.g. *P2ry12, Tmem119, Cx3cr1*) and have lower expression in EC *App* KI microglia compared to WT (Fig. 3D–G). Cluster B microglia, however, mostly originate from EC *App* KI samples (86.4%; Fig. 3B) and resemble DAMs identified in previous publications. A further 11.6% of cluster B microglia originate from DCN *App* KI samples, indicating the presence of small numbers of DAMs in the DCN. As expected, cluster B cells are strongly enriched for expression of gene module Mg.G2 (529 genes) which consists of genes associated with AD and expressed in DAMs (e.g. *Ank, Lpl, Csf1, Igf1, Itgax*; Fig. 3A,D–G) [30, 52]. Interestingly, expression of Mg.G2 (*App* KI-enriched) genes is also basally enriched in the DCN compared to EC (Fig. 3D, E). Overall, Mg.G2 genes show enrichment of GO biological processes linked to AD, including inflammatory response, kinase signalling cascades, cell surface signalling and regulation of angiogenesis (Fig. 3H).

Gene modules Mg.G3 and Mg.G4 were enriched in smaller populations of cells not identified as separate clusters. In particular, Mg.G3 (502 genes) is strongly enriched in the DCN compared to EC in WT mice (Fig. 3D, E). In the UMAP of microglia subpopulations, cells with strong expression of these genes formed a small spike protruding from the main cluster of homeostatic microglia and mainly comprised cells from DCN samples (Fig. 3C and F). GO analysis showed that this module is strongly enriched for interferon (IFN) pathways, defence response to virus, cytokine production, NFKB signalling, and autophagy (Fig. 3I). Individual genes in this cluster include *Sp100, Oasl2, Ifi204, Tlr1* and MHC class I antigen presentation genes *H2-K1, B2m,* and *Tap2* (Fig. 3G). The transcriptome profile for this population is most similar to “IRM” or “interferon-related” microglia reported by others [30, 32]. Overall, genes in this module were not differentially expressed between WT and *App* KI animals, despite the small number of IFN pathway DEGs we identified previously in the DCN (Fig. 3E). Thus, Mg.G3 provides a distinct regional signature differentiating DCN from EC that is independent of genotype. Finally, Mg.G4 (56 genes) is enriched for cell cycle genes specifically expressed in a small subpopulation of microglia, corresponding to the cycling/proliferating microglia subtype identified in previous publications (Additional file 1: Fig. S6 [30, 53]).

We found the differences in IFN-related gene expression between regions intriguing and considered that they may serve as an important molecular basis for the regional differences in Aβ pathology. To validate the enrichment of microglia expressing interferon response genes in the DCN, we performed in situ hybridisations probing for *Oasl2* and *Ifi204* transcripts in WT and *App* KI microglia in 7-month-old mice. Using *Hexb* as a marker of microglia, we quantified the expression of *Oasl2* and *Ifi204*, finding significant increases in the DCN compared to EC in both WT and *App* KI mice (*Oasl2*: *p* = 0.0037; *Ifi204*: *p* = 0.0085; Fig. 3J–M) but no significant differences between genotypes in either region. Overall, the density of *Hexb*-positive microglia was similar in EC and DCN in WT mice but significantly elevated in *App* KI mice in the EC, reflecting inflammation associated with Aβ pathology (Additional file 1: Figs. S7A-B). In addition, we observed large *Hexb*-positive microglial aggregates (> 100 µm^2^) in *App* KI tissues which are likely associated with plaques (Additional file 1: Fig. S7A). Importantly, we confirmed that the white matter surrounding the DCN has a lower density and proportion of *Oasl2/Ifi204/Hexb*-positive microglia compared to the DCN (Additional file 1: Fig. S7C-E), and that enrichment of *Oasl2/Ifi204*-positive microglia is specific to the DCN.

Overall, our snRNAseq results show that the DCN is enriched for a subtype of microglia expressing interferon response genes and has fewer of the AD-associated microglia subtype strongly enriched in the EC in 7-month-old *App* KI mice and previously described by others in AD mouse models. Our analysis of co-expressed gene modules successfully identified not only disease-related signatures, but also regional differences in glial transcriptional identities.

### Multiple glial populations in the DCN, but not EC, are enriched for gene expression associated with innate immunity and cytokine production

We next investigated whether other major glial cell types exhibit distinct regional transcriptomic signatures similar to the cytokine-enriched state found in DCN microglia. We reasoned that microglial reactivity may contribute to, or be otherwise associated with, alterations in gene expression in multiple cell types in the DCN, which may collectively affect vulnerability to plaque pathology.

**Fig. 4:**
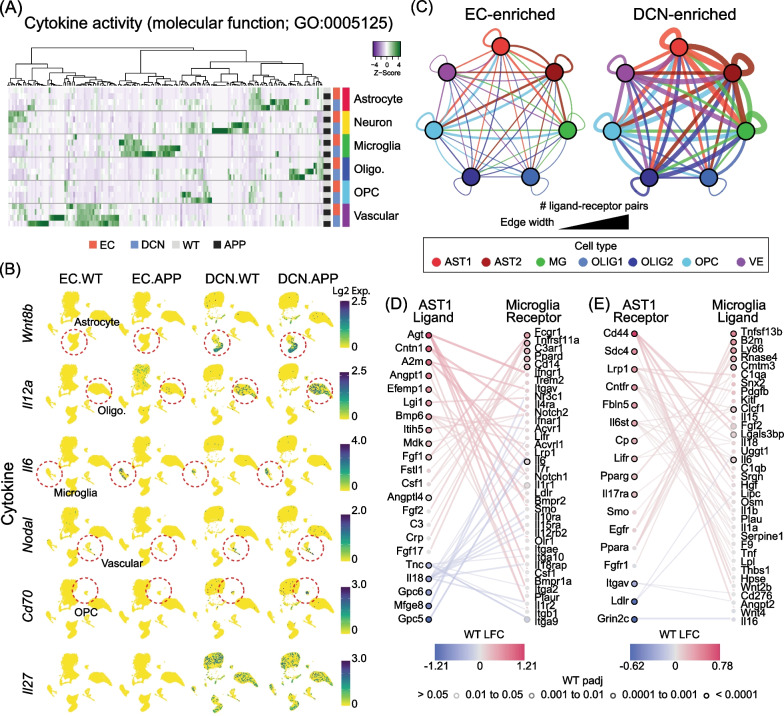
Multiple cell types in the DCN contribute to a cytokine-enriched microenvironment. **A** Heatmap showing pseudo-bulk average expression of ‘cytokine activity’ genes (GO:0005125) for each major cell type in each condition. **B** UMAPs of expression of representative DCN-enriched cell-type-specific cytokines. Colour scale indicates log-normalised expression. **C** Enrichment of ligand–receptor interactions associated with GO biological processes ‘immune system process’ (GO:0002376) and ‘inflammatory response’ (GO:0006954) in WT EC compared to DCN. Nodes represent glial cell types and edges represent ligand–receptor pairs with greater combined expression in each region. Edge thickness indicates number of pairs and edge colour indicates ligand cell type. **D** Top 50 ligand–receptor interactions associated with immune and inflammatory pathways between AST1 ligands and microglia receptors. Node colour and border indicate differential expression in WT DCN vs EC. **E** As in **D** for AST1 receptors and microglia ligands

We first investigated whether cytokine signalling is broadly enhanced in the DCN by analysing pseudo-bulk expression of genes associated with cytokine activity (GO:0005125). Strikingly, a large majority of cytokines detected by snRNAseq have higher expression in the DCN than the EC. Not only did we find strong expression of cytokines in DCN microglia (*Il6, Tnfsf13b)*, but we also found distinct subsets of cytokines enriched in astrocytes (*Wnt8b, Csf2, Flt3l)*, oligodendrocytes (*Il12a, Il17b, Cxcl17*), OPCs (*Cd70*) and vascular cells (*Nodal),* as well as cytokines enriched in multiple cell types in the DCN (*Il27*, *Il10*, *Il4;* Fig. 4A, B). Importantly, we also detected a subset of cytokine mRNAs associated with the disease phenotype that are primarily enriched in microglia in the EC of *App* KI mice (Fig. 4A).

We hypothesised that the enrichment of unique subsets of cytokines within different cell populations in the DCN is maintained by binding of secreted cytokines to receptors from multiple glial types. To test this idea, we compared ligand–receptor interactions in WT DCN and EC using a previously published database [54]. In agreement with our hypothesis, ligand–receptor interactions associated with immune and inflammatory processes were strongly enriched in the DCN between all glial types, with the greatest numbers of interactions between astrocyte subtypes (AST1-AST2), and astrocytes and microglia (AST2-MG and AST1-MG; Fig. 4C). Closer examination reveals a diverse and complex intercellular immune signalling network, with enrichment of both ligands and receptors found across all glial types, as evidenced by the top interactions between AST1 and microglia (Fig. 4D, E). We also observed many ligands with the potential to interact with receptors on multiple cell types, such as *Tnfsf13b*, a ligand that is enriched in DCN microglia and has receptors expressed in astrocytes (*Itga7, Ppara*), OPCs (*Tnfrsf13c, Itgb1, Ppara*), endothelial cells (*Itgb1*), and microglia (*Tnfrsf13b, Tnfrsf17, Itgb1*; Fig. 4D, E).

**Fig. 5:**
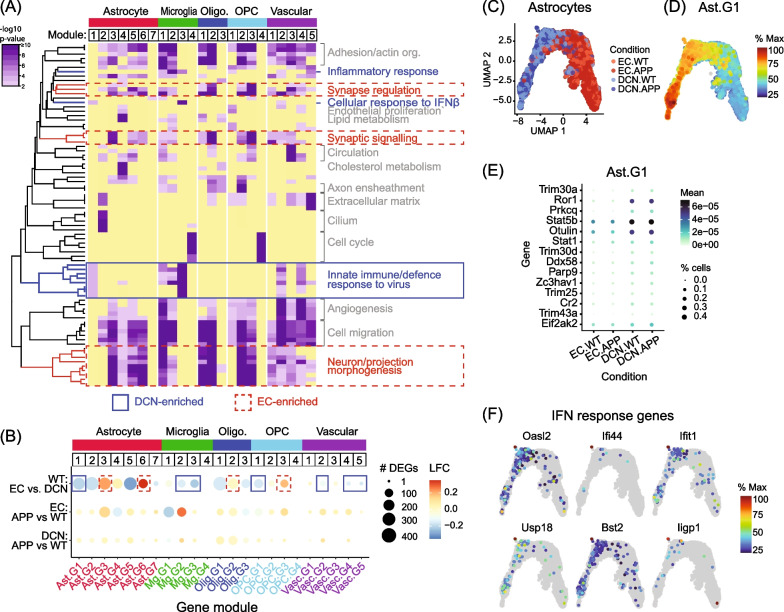
Co-expressed gene modules in multiple glial cell types linked to cytokine-enriched state. **A** Heatmap of top enriched biological processes within modules of co-expressed genes in each major type of glia (log_10_ *p*-value; *p* < 0.01). Clusters of related GO terms are labelled, with processes associated with EC-enriched (red) or DCN-enriched (blue) modules further highlighted. Yellow boxes represent non-significant expression. **B** Dot plot summarising modules of co-expressed genes contributing to transcriptional diversity in each major non-neuronal cell type. Dot size indicates number of DEGs in each module and dot colour indicates average LFC for three DE tests: WT.EC vs. DCN, EC.APP vs. WT, DCN.APP vs. WT. Boxes indicate DCN-enriched (blue) or EC-enriched (red) modules. **C** Astrocyte subcluster UMAP indicating cells from each condition. **D** Astrocyte subcluster UMAP showing expression of module Ast.1 as a percentage of maximum expression. **E** Dot plot showing expression of top DEGs associated with innate immune responses, cytokine production and NFKB signalling in Ast.1 across conditions. Dot size indicates percentage of cells in each condition expressing each gene and dot colour indicates mean log_2_ expression. **F** UMAPs showing expression of IFN response genes in astrocyte subpopulations as a percentage of maximum expression

To further characterise the differences between the DCN and EC transcriptomes in *App* KI and WT mice, we performed subcluster analyses for all major types of glia in our dataset: astrocytes, oligodendrocytes, OPCs (combined with COPs), and vascular cells, in addition to microglia described above. For each type of glia, we identified modules of co-expressed genes associated with cell subpopulations (Additional file 1: Fig. S8) and combined these data with the previously generated DEGs between regions and genotypes (see Fig. 2). We then analysed enriched GO biological processes in each module and summarised the information in a table and a heatmap for the top significant terms per module (Fig. 5A, B). From there, we identified subsets of genes within modules involved in specific processes of interest and investigated their expression patterns in more detail.

Similar to the enrichment of cytokine activity genes, we identified immune-related processes enriched across multiple cell types. For example, module Ast.G1 genes were more strongly expressed in WT DCN than EC (Fig. 5B–D), with top enriched genes involved in defence response to virus, cytokine production and response to IFNβ (DCN-enriched; Fig. 5A). Among the immune-related genes in Ast.G1 are *Trim30a, Trim25, Stat1, Stat5b, Prkcq, Ror1, Otulin and Ddx58* (Fig. 5E), as well as a set of interferon response genes (e.g. *Oasl2, Ifit1, Bst2, Usp18*, *Iigp1*) that were particularly enriched in a small subpopulation of DCN astrocytes (Fig. 5F). Besides astrocytes, we also identified two modules in vascular cells—Vasc.G2 and Vasc.G4—enriched for genes associated with the innate immune system, virus defence and NFKB activity (Figs. 5A-B and Additional file 1: Fig. S9). Importantly, both modules have higher mean expression in WT DCN compared to EC, as well as being enriched for DEGs upregulated in *App* KI in vascular cells in both regions (Fig. 5B and Additional file 1: Fig. S9). In addition to the cytokine signatures in the aforementioned glial subtypes, we also identified enrichment of smaller subsets of genes linked to the negative regulation of inflammation and/or of cytokine production in modules that had higher expression in DCN OPCs (OPC.G1), oligodendrocytes (Olig.G3) and vascular cells (Vasc.G5; Fig. 5B and Additional file 1: Fig. S9).

Apart from DCN-enriched modules, we noted that several gene modules with higher expression in the EC were primarily enriched for genes linked to synaptic signalling, regulation of synapse assembly and ion transport (EC-enriched; Ast.G3, Ast.G6, OPC.G3, Olig.G2; Fig. 5A, B). In astrocytes, both Ast.G3 and Ast.G6 modules are enriched in the EC of WT mice and include genes involved in receptor-mediated signal transduction and regulation of neuron differentiation and migration, as well as cell adhesion molecules and growth factors (Fig. 5A, B).

Overall, our snRNAseq data show differential expression of multiple pathways between DCN and EC glia that are relevant to disease processes. Most strikingly, the DCN is basally enriched for genes involved in cytokine production, interferon and NFKB signalling pathways, MHC class I antigen processing and inflammatory responses across different glial subtypes. As inflammatory pathways have been strongly linked to AD pathology, we speculate that the immune microenvironment of the DCN may contribute to the enhanced resistance to AD pathology.

### Tissue extracts from the DCN are enriched in cytokines

Changes in the transcriptome do not always correlate at the protein level [55]. To confirm that upregulated expression of cytokine mRNAs corresponds to elevated protein levels in the DCN, we performed a ProcartaPlex immunoassay on brain lysates. We selected a broad range of cytokine and chemokine targets from snRNAseq analysis that were enriched in the DCN compared to EC in a variety of cell types, as well as a subset enriched in *App* KI cortical microglia. We isolated brain tissue from WT and *App* KI mice (7-month-old males; *N* = 5 for each genotype), with EC and DCN isolated from one hemisphere, and cerebral cortex and cerebellum from the opposing hemisphere of the same subjects.

**Fig. 6:**
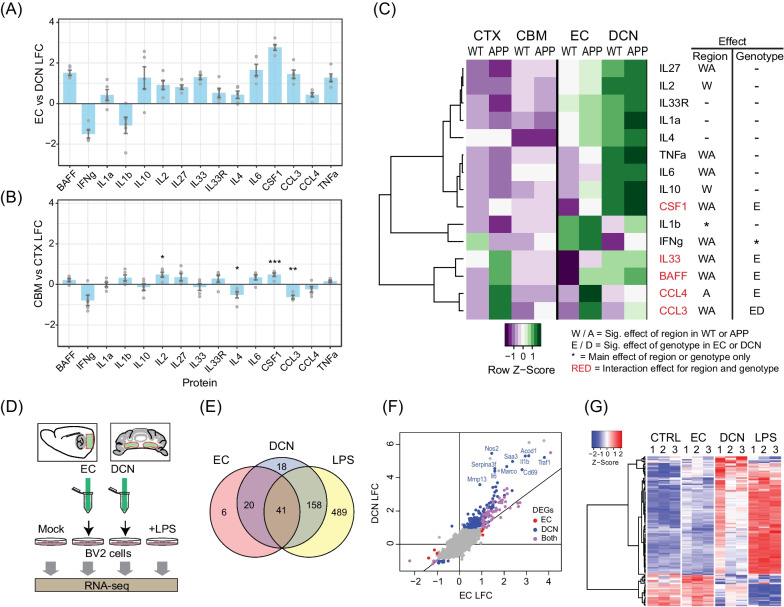
DCN is basally enriched for cytokine proteins and homogenates induce an inflammatory response in a cultured microglia cell line. **A**, **B** Differences in cytokine protein expression between regions in WT mice measured by multiplex array (*N* = 5). **A** DCN vs. EC. **B** Cerebellum (CBM) vs. cortex (CTX). **C** Heatmap of expression z-scores from multiplex cytokine array for each protein across regions and genotypes for WT and *App* KI mice. Two-way ANOVAs were performed to test for main effects of region and genotype and interaction effects, followed by Tukey HSD post hoc tests (*p* < 0.05). **D** Schematic of experiment stimulating cultured BV2 microglia with homogenates of EC or DCN, LPS (positive control) or DPBS (negative control), followed by RNA extraction after 4 h. **E** Venn diagram of the number of differentially expressed genes (DEGs) in each stimulation condition compared to control (LFC >  ± 1, FDR < 0.1). **F** Log_2_-fold changes in gene expression in EC and DCN conditions compared to control, with DEGs in each condition indicated by colour. Top DCN-specific DEGs are labelled. **G** Heatmap showing differential expression in DCN vs. EC (*p* < 0.01, 115 genes) across all samples

Overall, our array showed strong enrichment of multiple cytokines in WT DCN compared to EC: BAFF (TNFSF13B), IL10, IL2, IL27, IL33, IL6, CSF1, CCL3 and TNFα (Fig. 6A and Additional file 1: Fig. S10A). Most strikingly, we found more than sixfold higher expression of CSF1 and approximately threefold enrichment of BAFF, IL10, IL6 and TNFα in the DCN relative to EC (Fig. 6A and Additional file 1: Fig. S10A). There was also significantly lower expression of IFNγ in WT DCN compared to EC, although concentrations for all three interferons α, β and γ in our samples are low, with IFNα and IFNβ below the limits of quantification (data not shown). Surprisingly, extracts from the whole cerebellum are not similarly enriched for cytokine expression compared to cortex, with only CSF1 and IL2 showing significant but mild enrichment, while IL4 and CCL3 are depleted (Fig. 6B and Additional file 1: Fig. S10B). A direct comparison of cytokine concentrations between DCN and cerebellum indicates that the enrichment in DCN also holds true relative to the cerebellum (Fig. 6C and Additional file 1: Fig. S10). Even though CSF1 is reportedly a key upstream regulator of cerebellar microglial gene expression and function [56], our array data suggest that CSF1 ligand enrichment is much higher in the DCN compared to the whole cerebellum as well as the EC.

Comparing cytokine profiles between genotypes in each region, we found strong enrichment of specific cytokines in the EC and cortex of *App* KI mice compared to WT (CSF1, BAFF, IL33, CCL3, CCL4), with overall greater expression changes in the EC than in the cortex (Fig. 6C and Additional file 1: Fig. S10). The largest changes in expression were observed for CCL3 and CCL4, both of which are chemokines that are heavily expressed in inflamed tissues and upregulated in AD [57]. CCL3 was also the only significantly upregulated analyte in the DCN and cerebellum of *App* KI mice, with a much smaller induction than in the EC/cortex (Fig. 6C). Overall, changes in *App* KI-responsive cytokine levels in the EC/cortex are much higher than in the DCN/cerebellum, in line with the differences in AD-associated pathology in these regions.

In summary, the cytokine immunoassay data align with our transcriptome analysis and confirm that the DCN is enriched for inflammatory cytokines relative to the cortex and the cerebellum. More importantly, the basally enriched cytokine profile in the DCN is distinct from that in the EC and cortex induced by *App* KI pathology both in identity and response magnitude, suggesting a mechanistic distinction between cytokine expression in pathological states and in the cerebellar niche.

### DCN protein homogenates induce inflammatory gene expression in cultured microglia

We hypothesised that enriched cytokines in the DCN microenvironment contribute to the inflammatory gene expression profiles of DCN microglia and thus might induce a similar response in cultured microglia. To test this hypothesis, we treated BV2 microglia cell lines with DCN or EC protein homogenates, or LPS as a positive control for inflammatory response gene induction (Fig. 6D). We then analysed RNA expression 4 h post-stimulation, the peak timepoint of LPS-induced inflammatory gene expression [58]. To enrich for extracellular secreted proteins and to prevent effects of extraction buffer components on cell cultures, tissues were gently homogenised in DPBS on ice in the absence of detergent and protease inhibitors.

Using RNAseq (*N* = 3), we first identified DEGs in BV2 microglia in each condition compared to controls. DCN homogenates significantly altered expression levels of 237 genes. Of these DEGs, 80% were also regulated in response to LPS treatment (Fig. 6E). In comparison, we found significantly fewer DEGs with EC homogenates, of which all but six were shared with DCN and 41 were found in all three conditions. Strikingly, no DEGs were exclusively shared by EC and LPS conditions (Fig. 6E). STRING analysis of the DEGs altered in the DCN and LPS conditions, but not with EC homogenates, shows strong enrichment of ‘immune response’ (FDR = 9.91 × 10^–32^), ‘inflammatory response’ (FDR = 1.03 × 10^–18^), and ‘response to cytokine’ (FDR = 8.75 × 10^–26^; Additional file 1: Fig. S11). Inflammatory genes strongly upregulated in response to DCN but not EC homogenates include *Il1b, Il6, Nos2, Cd69 and Traf1* (Fig. 6F). In general, DEGs that responded more strongly to DCN homogenates were altered in the same direction as LPS-treated samples, albeit at a lower magnitude—which is to be expected as LPS triggers a strong immune response in BV2 cells (Fig. 6G) [58].

To validate some of the RNAseq data, we performed qPCR of selected inflammatory markers (*Il6, Il1b, Tnf, Csf1, H2-K1, Ifi204* and *Tlr1*) for BV2 cultures stimulated with paired EC and DCN homogenates (*N* = 8). We found significantly higher expression induced by DCN homogenates for *Tnf* (*p* < 0.001) and *Csf1* (*p* = 0.01; Additional file 1: Fig. S12), while *Il1b, Il6, H2-K1* and *Tlr1* all had higher mean expression in the DCN condition with p-values between 0.05 and 0.1. Overall, even though there is variation at the individual gene level, there is a significant main effect of input tissue, with expression of the eight inflammatory mRNAs tested being significantly higher for DCN than EC homogenates (*p* = 0.0162; Additional file 1: Fig. S12). We believe that the mild detergent-free extraction protocol and absence of protease inhibitors resulted in low protein concentration from the homogenates which blunted the inflammatory response in BV2 cells while increasing inter-sample variation. Nevertheless, the broader observations from the BV2 experiments align with our RNAseq data and immunoassays showing cytokine enrichment in the DCN and that DCN homogenates can induce an enhanced inflammatory response in BV2 microglia compared to EC homogenates.

### CSF1R inhibition alters inflammatory cytokine levels while further reducing DCN plaque abundance

We have identified a subpopulation of microglia expressing IFN response genes as part of a cytokine-enriched microenvironment in the DCN. Given that microglia secrete a variety of cytokines and are known mediators of neuroinflammation, it is unclear to what extent the DCN-microglia population is responsible for establishing the unique niche, and whether that contributes to the low plaque pathology observed in the region. To address this question, we performed a microglia depletion study to determine the impact not only on plaque deposition, but also on cytokine expression in the EC and DCN. While multiple microglia depletion studies have been conducted in AD mouse models to investigate the impact on cortical plaque pathology/abundance, none have been reported for the cerebellum or DCN as far as we are aware. One straightforward assumption would be that a loss of microglia would also reduce the expression of microglia-expressed cytokines in the DCN. This could potentially change the expression profiles and functions of other cell types and destabilise the microenvironment, which may then modulate plaque deposition. However, we are aware that loss of the DCN IFN microglia may also directly alter plaque deposition or clearance. Nevertheless, as a first approach to modulate the DCN immune environment, we used the CSF1R inhibitor PLX5622, a small molecule inhibitor that can be delivered non-invasively and has been repeatedly demonstrated to robustly deplete microglia [59].

**Fig. 7:**
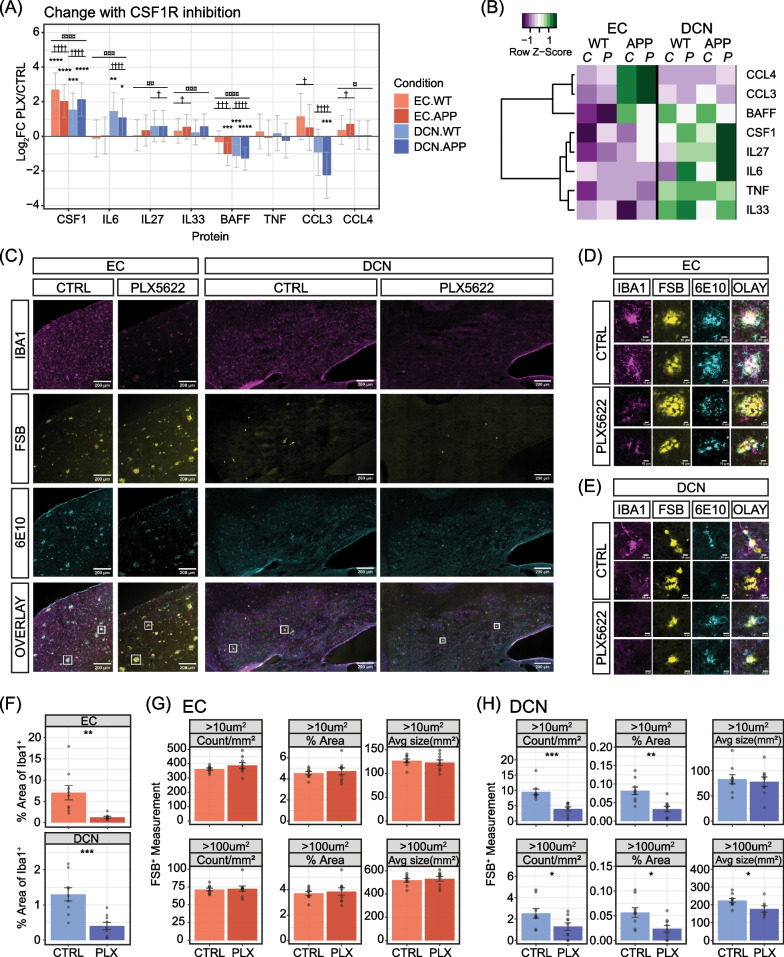
Microglia depletion by CSF1R inhibition reduces Aβ plaque abundance in the DCN and alters cytokine expression. **A**, **B** Multiplex array of cytokine protein expression in the EC and DCN. EC and DCN sections from *App* KI mice were fed chow containing PLX5622 or an inert dye (Control) for 60 days beginning at age 4 months. **A** Bar chart of log_2_ mean differences in cytokine expression between PLX5622 and control conditions for WT and *App*^*NL−G−F*^ mice in the EC and DCN. Error bars indicate 95% confidence intervals and stats were calculated using three-way ANOVAs followed by Tukey’s HSD post hoc tests. Significant differences are indicated for main effect of treatment (¤), effect of treatment in each region (†) and effect of treatment in each region for each genotype (*). * p < 0.05, ** p < 0.01, *** p < 0.001, **** p < *0.0001.* **B** Heatmap showing expression z-scores for each protein across all conditions. **C** Immunohistochemistry on brain sections was performed for soluble Aβ (α-6E10: cyan), microglia (α-IBA1: magenta), as well as fibrillar Aβ (FSB: yellow). Images were taken at 20X magnification. Scale bar 200 µm. **D**, **E** Examples of individual plaques from subfigure (**C**) in the EC (**D**) and DCN (**E**). Scale bar at 10 m. **F** Quantification of IBA1^+^ microglia coverage in the EC and DCN of *App* KI mice fed with PLX5622 and control chow, showing significant depletion in both regions (*N* = 9 subjects, *n* = 3 sections/subject). **G**, **H** Quantification of number, total area and average size of FSB^+^ plaques in EC (**G**) and DCN (**H**), for two different plaque size thresholds (> 10 µm^2^ and > 100 µm^2^; *N* = 9 subjects, *n* = 3 sections/subject)

Using a published protocol [60], we initiated feeding with 4-month-old mice and harvested the EC and DCN from WT and *App* KI mice after 2 months of treatment with PLX5622 or control diet (*N* = 5 for each group). We then repeated the multiplex immunoassay with a subset of the cytokines we found to be differentially expressed between regions or genotypes above (Fig. 6A–C). As well as largely confirming our previous findings of enrichment for select cytokines in DCN and in *App* KI cortex, the array showed substantial alterations following treatment (Fig. 7A and Additional file 1: Fig. S13). Contrary to our expectations that loss of microglia would dampen expression of cytokines, we observed mixed results upon CSF1R inhibition by PLX5622. In particular, CSF1 levels which we previously showed to be highly enriched in the DCN, are further enhanced after PLX5622 administration in both regions for both genotypes (Fig. 7A, B). Furthermore, we found that in the DCN, levels of IL6 and IL27 were also significantly increased, while BAFF and CCL3 were reduced. Intriguingly, changes in the DCN do not correlate with the EC as levels of IL6 and IL27 were unaltered in the EC, whereas IL33, CCL3 and CCL4 levels were elevated (Fig. 7A, B). Of the cytokines we assessed, only TNF was not affected by PLX5622 administration in either region. Overall, our study reveals that PLX5622 treatment not only alters cytokine expression profiles in the brain, but potentially does so in a region-specific manner.

Next, we assessed the efficiency of PLX5622-mediated microglia depletion in *App* KI mice by immunohistochemistry. We found that the area covered by IBA1-positive microglia in the EC was depleted by 82% on average (p = 0.00380), while coverage in the DCN was depleted by 70% (p = 6.97 × 10^–4^; Fig. 7C and F). In PLX5622-treated WT mice, microglial depletion also occurred with high efficiency (EC: CTRL vs. PLX 83%, *p* = 0.0003; DCN: CTRL vs. PLX 92%, *p* = 0.0264; Additional file 1: Fig. S14). Overall, the quantification of IBA1-positive microglia confirms significant depletion in the EC and DCN after PLX5622 treatment.

Finally, we examined whether plaque deposition is differentially impacted in the EC and DCN in *App* KI mice after PLX5622 treatment. For this, we used FSB and α-6E10 to detect fibrillar and diffuse plaques, respectively. In the EC, we found no significant differences in FSB-positive plaque count, total area or average size between treatment groups (Fig. 7G). In the DCN however, we found that FSB-positive plaques were further reduced following PLX5622 administration from an average count of ~ 9.5/mm^2^ in control subjects, to ~ 4/mm^2^ in treated subjects for plaques at a threshold of > 10 µm^2^ (*p *= 1.75 × 10^–4^), and from 2.5/mm^2^ to 1.3/mm^2^ at a threshold of > 100 µm^2^ (*p* = 0.0425; Fig. 7C–E and H). In addition to plaque counts, the total area was also reduced (> 10 µm^2^: *p* = 1.31 × 10^–3^; > 100 µm^2^: *p* = 0.0127) and average plaque size was lower for plaques with a size threshold of > 100 µm^2^ (*p* = 0.0352; Fig. 7H). Surprisingly, no significant differences were observed for diffuse plaques with α-6E10 staining in either region (Additional file 1: Fig. S15).

Overall, our PLX5622 treatment in mice successfully depleted the majority of microglia in EC and DCN, but with unexpected changes in cytokine profiles. We conclude from our detailed plaque measurements that PLX5622 treatment had minimal effects on amyloid pathology in the EC, but robustly depleted fibrillar plaques in the DCN. Finally, our experiments underscore the complex relationship between microglia and cytokine expression which must be taken into account when assessing plaque pathology.

## Discussion

### Microglia states in WT and AD mice across brain regions

In this report, we leveraged the disparity in amyloid pathology between EC and DCN to identify molecular signatures and signalling pathways that may contribute to these differences. We profiled shared and distinct pathways impacted by Aβ pathology in glia and also identified a subpopulation of microglia that is enriched in the DCN but independent of disease-associated states. These DCN-enriched microglia express genes closely linked to innate immune and type I interferon responses, similar to the cerebellar microglial population previously profiled via microarrays by Grabert and colleagues [61]. Notably, the transcriptional signature of DCN-enriched microglia also closely resembles microglial states identified in the cerebral cortex and previously reported in AD phenotypes as interferon-response or -responsive microglia [30, 32, 62, 63]. The proportion of these interferon-responsive microglia relative to homeostatic or AD-associated microglia in the cortex is small [30], and while they share overlapping patterns of gene expression with the DCN-enriched microglia, whether they are functionally equivalent remains to be determined. However, accumulating evidence indicates that overall, forebrain and cerebellar microglia are both morphologically and functionally distinct [56, 61, 64, 65]. In the cerebellum, elevated levels of CSF1 and CSF1-CSF1R interaction drives microglial identity and function [56], with cerebellar but not cortical microglia existing in a basally more “reactive” state and playing crucial roles in neuronal attrition and clearance [64]. Our profile of the DCN transcriptome, cytokine immunoassay and BV2 induction experiments all support the premise that the microglia in DCN exist in a unique cytokine-enriched state under wild-type conditions (Additional files 2 and 3).

### Different glial populations contribute to cytokine-enriched niche in the DCN

A broader analysis of the single-nuclei transcriptome indicates that the cytokine-enriched state in the DCN is not limited to microglia but extends to other cell types in the region. It is striking that glia, neurons and vascular cells all show broad elevation of cytokine expression in the DCN relative to the EC, even in the absence of plaque pathology. Importantly, not all cytokines have elevated expression in the DCN with some showing similar levels to EC, while others are depressed. This selective regulation is also not exclusive to either pro- or anti-inflammatory cytokines, as examples of both groups can be found up- or down-regulated across multiple cell types. It is important to note that our transcriptome and immunoassay data also show disease-specific changes in cytokine expression in the EC, and to a much lesser extent in the DCN. Curiously, the cytokine immunoassays of whole cerebellum relative to cortex show only a mild enrichment in a smaller subset of the cytokines. One possibility—as the majority of studies have examined cerebellar properties as a whole—is that the DCN could serve as a primary source of cytokine enrichment in the cerebellum [56, 61, 64].

Intriguingly, the PI3K–AKT/Rho GTPase pathway, which is enriched in *App* KI DCN astrocytes and oligodendrocytes, can be activated by cytokines [66–68] and is known to modulate different aspects of brain inflammation by regulating glial cell reactive states and crosstalk between populations [69–71]. Given that a sizeable proportion of DEGs in the DCN is found in oligodendrocyte subtypes and a subset of these are linked to PI3K–AKT/Rho GTPase activity, future studies will be aimed at exploring if the signalling pathway is preferentially activated in these cells, and whether that is directly tied to cytokine activity in the DCN and reduction of plaque pathology.

### CSF1R inhibition reduces plaque abundance in the DCN

Given our observations, we wanted to further explore the contribution of microglia to establishing the cytokine-enriched microenvironment and low plaque abundance in the DCN. Since a sizeable subset of cytokines are detected in DCN microglia, we hypothesised that these cytokines may play a role in regulating the expression and secretion of other cytokines in neighbouring cell types. In theory, if DCN microglia function as a master regulator for coordinated cytokine expression across cell types, then prolonged microglia depletion should dampen the overall cytokine-enriched state in the DCN and alter parameters of plaque formation and/or growth. We therefore chose to administer PLX5622 to deplete microglia as it has been proven effective in eliminating the vast majority of microglia despite reports indicating varying effects of the drug on amyloid pathology depending on the age of onset and length of drug treatment, inter-batch efficacy of the drug, brain region examined, AD rodent model used in the study and plaque detection methodology [59, 60, 72–74].

Contrary to our expectations, PLX5622 treatment in *App* KI not only failed to dampen cytokine expression in the DCN, it enhanced expression of a subset of cytokine targets we tested in the immunoassays. The increase in cytokine levels is most striking in DCN, but also present to a smaller extent in the EC. Notably, not all cytokines we tested are impacted by PLX5622 treatment. One interpretation of this result is that loss of cytokine expression in the microglia is compensated by increased expression in other cell types, with the compensation strongest in DCN due to the robust pre-existing pattern of expression across multiple cell types. Indeed, our data provide an explanation for observations by Vichaya and colleagues (2020) where microglial depletion by genetic and pharmacological means failed to attenuate, but instead exacerbated, LPS-induced sickness in rodents [75]. Another possibility is that long-term treatment with PLX5622 has been shown to give rise to a subpopulation of IBA1^+^/TMEM119^−^ treatment-resistant microglia [76]. Presumably, the remaining microglia could be induced to express and secrete more cytokines to compensate for the loss. However, it is unlikely that a small fraction of microglia remaining in the DCN after depletion could be induced to express higher levels of cytokines compared to the entire microglial population at basal state.

In contrast with our original hypothesis where we anticipated an increase in plaque pathology upon lowering the cytokine-enriched state in the DCN, we instead observed a significant reduction in both number and size of fibrillar plaques in the DCN of PLX5622-treated animals. This aligns with several studies which show that modulating the inflammatory response in the brain differentially impacts the deposition of dense core and diffuse amyloid plaques [77, 78]. Ironically, the increase in cytokine expression after drug treatment remains entirely consistent with our hypothesis that a cytokine-enriched environment is playing a role in reducing plaque deposition in the DCN. What is less clear are the candidates that drive this change. Many of the cytokines targeted in the immunoassay such as IL10, IL33 and IL6 have altered expression in post-mortem tissues and animal models, with some capable of influencing AD pathology [77, 79–83]. It is important to reiterate that while individual cytokine expression has been shown to modulate amyloid plaque pathology, it remains possible that the combined action of multiple cytokines—each with varying effects on inflammation and cell states—will influence plaque deposition in a complex microenvironment such as the DCN.

Perhaps rather fortuitously, one of the unintended consequences of PLX5622 treatment is the decoupling of microglial presence in the DCN from the expression of cytokines and phagocytosis of plaques. This eliminates the idea that microglial presence alone is responsible for establishing the cytokine-enriched state in the DCN. It is also unlikely that enhanced microglia-mediated phagocytosis is the main mechanism for the reduced fibrillar plaque pathology in the DCN. However, our result does not rule out the possibility that microglial involvement is required at the initial stages of plaque pathogenesis [59, 84]. Due to the late appearance of amyloid plaques in the DCN, our PLX5622 treatment to eliminate microglia at four months of age may result in the failure of plaque compaction that disrupts the emergence of new plaques [59].

Instead, our data open up the possibility that a cytokine-mediated reduction in plaque pathology could be achieved indirectly via mechanisms that accelerate clearance of Aβ in the DCN. This could be accomplished through the glymphatic or vascular systems where it is well-known that cytokines can alter blood brain barrier integrity, vascular permeability and blood flow, and lymphatic drainage [85–88]. At least one report has also shown that synthetic Aβ injected in *App* KI mice is cleared more rapidly in the cerebellum than in the cortex [89]. Alternatively, the DCN microenvironment may recruit other myeloid populations or heighten phagocytic activity of other cell types [90–92]. At present, it is also unclear if the DCN microenvironment is playing a role in suppressing tau pathologies. While tau tangles in the cerebellum have only been reported in rare cases [93, 94], experiments where cytokine levels are manipulated in animals that express mutant tau in the DCN will prove to be insightful. Overall, while experiments with PLX5622 in *App* KI mice have generated new insights into the relationship between microglia, cytokine expression and amyloid pathology, future microglia depletion experiments must also consider changes in cytokine expression from other cell types in response to the depletion mechanism when assessing the impact on AD pathology.

In this study, only male mice were used, and we cannot rule out sex-specific differences in AD pathology that exist between the different brain regions given that females often show higher levels of AD pathology [95]. Nevertheless, the absence of plaques in the DCN and, to a lesser extent, in the cerebellum has been reported in post-mortem brains for both men and women [5, 96]. Certainly, from a behavioural standpoint, the lack of pathology in the cerebellum correlates well with no significant loss of locomotor skills during early stages of the disease, unlike neurodegenerative disorders where cerebellar degeneration is detected [97]. Of note, the DCN in mice has been found to regulate other behavioural outputs including those that control satiety [22] and additional work should be done to examine whether these outputs are affected by AD pathology.

It is important to note that even though amyloid plaques are sparser in DCN than EC, several lines of evidence indicate that the expression of full-length APP in both brain regions is comparable. First, our transcriptome data indicate that overall *App* expression across all cell types is not significantly reduced in DCN (Additional file 1: Fig. S16). Furthermore, a recent study showed that full-length APP protein expression in *App* KI cerebellum is comparable to WT animals [89]. Taken together, we do not think that altered APP expression in EC-DCN is a contributing factor to the differences in pathology. In addition, we also examined a selection of genes directly involved in APP processing and generation of Aβ [98–101]. In our dataset, the expression of these genes in the DCN is comparable to or slightly higher than in EC. However, there are many genes associated with the processing and trafficking of APP and we cannot entirely rule out that a subset of these genes with altered expression may end up contributing toward differences in plaque phenotypes [102].

Overall, our snRNAseq profiles of glia in the EC and DCN reveal the presence of a cytokine-enriched microenvironment unique to the DCN that is likely established through the collective expression of a variety of cytokines across multiple cell types. We show that the DCN milieu can induce an inflammatory transcriptional response and by manipulating this niche, we can alter deposition and growth of amyloid plaques in the DCN.

## Methods and materials

### Animals

Adult male C57BL/6J WT and *APP*^*NL-G-F/NL-G-F*^ (*App* KI) mice were used in this study. All animals were housed with free access to food and water in the Animal Research Facilities in the Lee Kong Chian School of Medicine, Nanyang Technological University, Singapore. All procedures were approved by the Nanyang Technological University Institutional Animal Care and Use Committee (IACUC18095).

### Histology

Animals were anaesthetised with isoflurane before transcardial perfusion with cold 1X phosphate buffered saline solution (PBS) followed by 4% paraformaldehyde (PFA). Brains were extracted and post-fixed in PFA, then preserved in 30% sucrose for 2–3 days. The brains were then frozen in OCT medium (Sakura Finetek, 4583) on dry ice and stored at -80 ºC until sectioning. For the analysis of Aβ plaques from 3–12 months, brains were cryosectioned at 30 µm and mounted on Fisherbrand™ Superfrost™ Plus microscope slides then stored at –80 ºC. For PLX5622 feeding experiments, 30-µm sections were stored free-floating in cryoprotectant solution [30% ethylene glycol, 30% glycerol in 0.02 M phosphate buffer] at − 20 °C. For in situ hybridisation, tissues were cryosectioned at 12 µm and mounted on Fisherbrand™ Superfrost™ Plus microscope slides then stored at − 80 °C.

### Immunohistochemistry

For analysis of Aβ plaques and IBA1, tissues were washed with 0.1% PBST [0.1% Triton-X in PBS] three times for 5 min each. Tissues were then blocked with 3% horse serum in PBST for 2 h at room temperature before overnight incubation with primary antibodies at 4 °C. On the following day, sections were washed thrice, then incubated with secondary antibodies for 1 h at room temperature. Sections were washed, then incubated with DAPI for 5 min or FSB for 30 min, followed by a final wash before being mounted.

For comparison of 6E10 + plaques between regions from 3–12 months, single plane images were captured with a Zeiss AxioScan.Z1 slide scanner at 20X magnification and analysed using Fiji (NIH). First, region of interests (ROI)s were drawn, and the area was measured. To perform background correction, minimum and maximum intensity values were set manually for each section. Then the triangle algorithm was used for conversion to binary images, followed by detection of particles larger than 10 µm^2^ and 100 µm^2^. Full statistical test results are presented in Additional File 2.

### Antibodies and dyes

**Table:** 

Primary antibodies	Source	Dilution
Aβ (6E10; mouse)	BioLegend, 803001	1:1000
IBA1 (rabbit)	FUJIFILM Wako, 019–19741	1:1000

Secondary antibodies	Source	Dilution
Donkey anti-mouse IgG (H + L), Alexa Fluor™ 647	Thermo Fisher Scientific, A-31571	1:1000
Donkey anti-rabbit IgG (H + L), Alexa Fluor™ 555	Thermo Fisher Scientific, A-31572	1:1000

Dyes	Source	Dilution
Hoechst 33342	Thermo Fisher Scientific, H3570	1:1000
DAPI	Invitrogen™ D3571	300 nM
FSB staining solution	Sigma-Aldrich, 07602	1:5000

Dyes	Source	Dilution
Hoechst 33342	Thermo Fisher Scientific, H3570	1:1000
DAPI	Invitrogen™ D3571	300 nM
FSB staining solution	Sigma-Aldrich, 07602	1:5000

### Hybridisation chain reaction (HCR) RNA fluorescent in situ hybridisation

Tissues were pre-treated as follows: fixed with ice cold 4% PFA for 10 min, washed with 1X PBS three times for 3 min each, digested with Proteinase K [1 µg/mL proteinase K, 5 mM EDTA, 50 mM Tris pH7.5 in MilliQ-H_2_O] for 10 min, fixed with 4% PFA for 5 min, washed with 1X PBS three times for 3 min each, acetylated for 10 min [acetylation solution: triethanolamine (Sigma, 108-24-7) and acetic anhydride (Sigma, 411000) in MilliQ-H_2_O] and finally, washed with 1X PBS three times for 5 min each. Pre-hybridisation with hybridisation buffer (HB; Molecular Instruments, Inc.) for 60 min is carried out in humidified chamber at 37 °C. Following that, 2 pmol of HCR probes (Molecular Instruments, Inc.) in HB were added onto the slides with coverslips on for overnight incubation in humidified chamber at 37 °C. After 16 h of incubation, slides were washed with 5xSSCT [5 × SSC, 0.1% Tween 20 in MilliQ-H_2_O] three times for 15 min each and wash buffer (WB; Molecular Instruments, Inc.) for 30 min at 37 °C. Further two washes with 5xSSCT and amplification buffer (AB; Molecular Instruments, Inc.) were carried out at room temperature for 15 min each. The following amplification steps were performed according to manufacturer's protocol. Briefly, 6 pmol of hairpin in AB was added onto the slides and incubated overnight in the dark at RT. On the next day, three washes with 5xSSCT for 15 min followed by Hoechst staining was carried out. Sections were washed twice with 5 × SSCT for 5 min each before being for imaging. All HCR-FISH images were captured using Zeiss LSM810 Confocal Microscope System. Images were captured using 20X objective with five z-stacks of 1 µm and processed with Fiji (NIH). The z-stack images were projected using maximum intensity method before the ROI (i.e. EC or DCN) was cropped using the ‘freehand selection’ tool. Quantification of *Hexb* density was performed as follows: threshold was first applied to the *Hexb* channel using the in-built ‘Triangle’ algorithm and particles larger than 10 µm^2^ were selected. Density of microglia was calculated as the total area of *Hexb* particles divided by the area of ROI. For quantification of IFN response gene (IRG) signals within the same ROI, a 5% intensity threshold was used and pixels larger than 1 µm^2^ within the *Hexb* cells were selected. Percentage of IRG^+^/*Hexb*^+^ cells relative the total *Hexb*^+^ population was calculated. All data were analysed with GraphPad Prism 7. Full statistical test results are presented in Additional File 2.

### Microdissection of DCN and EC and nuclei isolation

Mice were anaesthetised with isoflurane before being decapitated. Brains were dissected on ice and rested in pre-bubbled slurry cutting solution [250 mM sucrose, 26 mM NaHCO_3_, 10 mM [D +] glucose, 3 mM myo-inositol, 2.5 mM KCl, 2 mM sodium pyruvate, 1.25 mM NaH_2_PO_4_·2H_2_O, 0.5 mM ascorbic acid, 1 mM kynurenic acid, 0.1 mM CaCl_2_ and 4 mM MgCl_2_] for 1 min. A downward cut across the midbrain was made to separate the cerebellum from the forebrain. The cerebellum was first sectioned at 300 µm in cold cutting solution using a vibratome [Leica VT-1200]. Approximately 4–5 sections were used for microdissection of the DCN (Additional file 1: Fig. S1) in chilled pre-bubbled artificial cerebrospinal fluid [ACSF: 10 mM [D +] glucose, 126 mM NaCl, 24 mM NaHCO_3_, 1 mM NaH_2_PO_4_·2H_2_O, 2.5 mM KCl, 0.4 mM ascorbic acid, 2 mM CaCl_2_ and 2 mM MgCl_2_]. For diagram of dissection, refer to Additional file 1: Fig. S1. EC dissections were performed on wetted filter paper on ice and dissected tissues were kept in chilled ACSF with continuous carbogenation for up to 10 min until homogenisation. For homogenisation, tissues were transferred to chilled homogenisation buffer [0.25 M sucrose, 25 mM KCl, 5 mM MgCl_2_, 20 mM Tricine, pH 7.8] in a Dounce homogeniser and homogenised with 10 strokes of the pestle. To lyse the samples, NP-40 was added to obtain a final concentration of 0.3% and samples were dounced with 5 strokes. Lysates (2 ml) were filtered using 40 µm FlowMi tip strainers and gently mixed with 4.6 ml 1.8 M sucrose cushion buffer [1.8 M sucrose, 10 mM Tris–HCl, 1.5 mM MgCl_2_, pH 6.9] then layered over a 3-ml sucrose cushion. Samples were spun in an ultracentrifuge at 30,000xg for 45 min at 4 °C. The supernatant was removed, and pellets were resuspended in 90 µl nuclei resuspension buffer [10 mM Tris–HCl, 0.25 M sucrose, 25 mM KCl, 1.5 mM MgCl_2_, 1.5 mM CaCl_2_, pH 6.9] before quantification.

### Single nuclei RNAseq and data analysis

The cDNA libraries were generated according to manufacturer’s protocol (10 × Genomics, Inc.). In brief, approximately 10,000 nuclei per sample were processed using the Chromium Single Cell 3’ v3 Gene Expression kit [103]. The libraries were sequenced on the NovaSeq6000 platform (NovogeneAIT Genomics).

### Pre-processing and dimensionality reduction

SnRNAseq data were first aligned to the mm10 pre-mRNA reference genome using CellRanger v3.1.0 with an expected cells value of 10,000. Downstream processing was performed using Seurat v4.1 [104]. First, all samples were processed individually. Barcodes with > 5% mitochondrial RNA were removed. UMIs were normalised by library size, multiplied by a scale factor of 10,000 and log transformed. Highly variable genes (*N* = 5000) were identified using the variance-stabilising transformation method. A shared nearest neighbour graph was constructed, and clustering was performed using the Louvain algorithm, followed by uniform manifold approximation and projection (UMAP) mapping into two-dimensional space. We used 45 principal components (PCs) for DCN and 60 PCs for EC with clustering resolution 0.7. Clusters of droplets with very low counts and numbers of features as well as a relatively high percentage of mitochondrial RNA, representing low-quality cells and/or ambient RNA, were removed. DoubletFinder v2.0.3 [105] was used to label droplets that may contain multiplets (parameters: 45 PCs, sct = false, GT = false, pN = 0.25).

SCTransform v2 [106] was applied to each sample, followed by SCT integration using 50 PCs and 3000 features. Dimensionality reduction was performed on the integrated dataset as described above at low (res. = 0.3, PCs = 30), medium (res. = 0.7, PCs = 50) and high (res. = 1.0, PCs = 70) resolutions and cluster labels were determined by marker gene expression [24]. Each major cell type (astrocytes, neurons, microglia, oligodendrocytes, OPCs and vascular cells) was then analysed using Monocle3 to classify cell subtypes. During these steps, further filtering was performed using marker genes and DoubletFinder to identify clusters of multiplets and low-quality cells. Final cell-type classifications were adjusted according to results of subcluster analyses. The final clusters were astrocyte 1, astrocyte 2, Bergmann glia, ependymal cells, excitatory neurons, Cajal Retzius cells, interneurons, microglia, peripheral immune cells, oligodendrocyte 1, oligodendrocyte 2, committed oligodendrocyte precursor cells, oligodendrocyte precursor cells, vascular endothelial cells, pericytes and leptomeningeal cells. Astrocyte subclusters broadly correspond to fibrous (higher expression of *Gfap*, *Kcnj3*, *Ablim2*, *Slc38a1*, *Rfx4* and *Dach1* in both regions; AST1) and protoplasmic (higher expression of *Gria2*, *Slc7a10*, *Slc6a11*, *Mgat4c* and *Kcnd2* in both regions; AST2) subtypes.

### Differential expression analyses

Differential expression analyses were performed using Seurat to apply the Wilcoxon rank sum test with Bonferroni correction on RNA counts for glia and vascular cell clusters with over 900 cells, comparing APP and WT genotypes in the EC and DCN, and EC and DCN regions in WT mice. DEG thresholds are adjusted *p*-value < 0.05, LFC >  ± 0.25, and expression in > 10% of cell population in either condition. All lists were combined, and a Venn diagram was plotted using the R package VennDiagram [107]. Filters to minimise contamination from ambient RNA were applied to each cell type as described below.

Genes that may be affected by contamination from the ambient pool were identified using the dropletUtils package [108, 109]. Ambient expression profiles were estimated for each sample from raw counts, with barcodes that had fewer than 500 UMIs classified as empty droplets. These profiles were used to calculate an upper bound for the proportion of expression counts contributed by the ambient pool for each gene in each cell population, using mitochondrial genes and lncRNAs associated with ribosomal RNA contamination (*Gm42418, Gm26917*) [110] as negative control genes. For each cell type, only genes for which the upper bound of ambient contamination was < 10% in at least one condition were included as DEGs to minimise false positives resulting from expression in other cell types. Furthermore, genes for which the upper bound of ambient contamination was > 10% in all conditions and > 50% in any condition were excluded from all downstream analyses for that cell type. The latter thresholds were chosen to exclude genes for which the majority of reads likely originated from the ambient pool, while retaining genes expressed in multiple cell types or highly expressed in abundant cell types (e.g. oligodendrocyte-enriched genes in oligodendrocyte subpopulations) as the upper bound of ambient contamination for such genes was often between 10 and 50%, as determined by examination of expression across cell types both in our dataset and in cell-type-specific mouse brain transcriptome databases [24, 111]. Differential expression test results are presented in Additional File 3.

A correlation matrix was plotted using the corrplot package [112]. Filtered gene lists for all glial cell types were combined and Pearson’s r was calculated for APP vs WT LFCs between all pairs of cell populations in each region. The proportion of shared DEGs was calculated relative to the total number of unique DEGs for each pair of cell populations. DCN-specific DEGs were then identified within each cell type and gene lists were combined for further analysis. Pathway analyses were conducted using the R package clusterProfiler [113] with the KEGG, WikiPathways and ReactomeDB databases [114–116], applying Benjamini–Hochberg correction for multiple comparisons (parameters: gene set size 10–1000, adjusted *p*-value < 0.05, *q* < 0.2). The protein–protein interaction network for DEGs was generated using STRING v11.5. Cytoscape v3.9.1 [117] was used to calculate node degree and plot the network with the Prefuse Force-directed layout.

Pseudo-bulk expression was calculated for major cell types (astrocytes, microglia, neurons, oligodendrocytes, OPCs and vascular cells) using Seurat RNA average expression scaled to 10,000 counts. Heatmaps display per-gene z-scores and hierarchical clustering was performed using Pearson’s correlation as the distance metric with the Ward D2 algorithm.

Analysis of ligand–receptor interactions was performed on the results of DE tests comparing WT DCN and EC using CCinx, in which edge weights are calculated as the sum of scaled LFCs [54]. Interactions were filtered to unique ligand–receptor pairs containing one or both genes annotated to the GO biological processes inflammatory response (GO:0006954) or immune system process (GO:0002376), including regulatory genes. A table was generated summarising the number of interactions with greater edge weights in each region between each pair of cell types and then plotted in Cytoscape.

### Subcluster analyses

Subcluster analyses for each major cell type were performed using Monocle3 [50, 51, 118]. First, 50 PCs were calculated, and cell transcriptomes were aligned using sample as the alignment group and total RNA counts plus percentage of mitochondrial reads as the residual model formula. UMAP co-ordinates were then calculated, and unsupervised clustering was performed by constructing a *k*-nearest neighbours (kNN) graph followed by Leiden community detection (k = 10 for vascular cells; *k* = 20 for all other cell types). The numbers of cell subclusters identified were astrocytes = 6, microglia = 2, oligodendrocytes = 5, OPCs = 2, vascular cells = 4. Spatial autocorrelation analysis was then performed using the Moran’s *I* test to identify genes that are differentially expressed across cell subpopulations in UMAP space. Genes with *q* < 0.05 that passed the filter for ambient contamination described above were then clustered into co-expressed modules (*k* = 5 for glia types with > 2 clusters; *k* = 10 for microglia and OPCs; maximum 4 components). The following modules with ≥ 50 genes were identified: astrocytes = 7, microglia = 4, oligodendrocytes = 3, OPCs = 4, vascular cells = 5. Gene modules are presented in Additional file 3. For each module, the degree of differential expression between conditions was determined using the results of DE tests conducted above: the number of DEGs (pooling unique DEGs across subtypes where applicable) and pseudo-bulk average expression for each contrast of interest.

GO analyses were performed to identify enriched biological processes (BP) in each module compared to a background list of expressed genes in that cell type using Fisher’s test implemented by the topGO package with minimum 5 annotated genes per term [119, 120]. To generate bar plots of top enriched terms, the weight01 algorithm was used to reduce redundancy between terms. Significant GO terms for each module are presented in Additional file 3. To compare top enriched processes across all modules, the results of unweighted GO tests were combined, and the resulting matrix was filtered to the top five enriched terms in each module with *p* < 0.01 and between 10 and 1000 annotated genes. Jaccard similarity coefficient was used to quantify gene set overlap and used as the distance metric for hierarchical clustering of GO terms using the Ward D2 algorithm. A heatmap was plotted displaying log10 p-value where *p* < 0.01, with the colour scale truncated at *p* = 1 × 10^–10^). Clusters of similar terms were labelled and highlighted manually.

### Cytokine immunoassays

EC was dissected as described above, while the cerebellum was coronally sliced into 1 mm sections using a brain matrix prior to dissection of the DCN in cold PBS using a scalpel. For the immunoassay that included cortex and cerebellum samples, EC and DCN were dissected from one hemisphere, while cerebellum and cortex (midbrain and hippocampus removed) were obtained from the other. For the remaining experiments, both hemispheres were combined. Tissues were snap frozen in liquid nitrogen and stored at -80 ºC until extraction.

Chilled cell lysis buffer [20 mM Tris–HCl, pH 7.4; 150 mM NaCl, 1 mM Na_2_EDTA, 1 mM EGTA, 1% Triton-X and cOmplete protease inhibitor] was added to tissues thawed on ice (500 µl/100 mg tissue) and. samples were homogenised with a micro-pestle (Bel-Art) followed by rigorous pipetting. Samples were centrifuged at 16,000 xg for 10 min at 4 °C and the supernatant was stored at − 80 °C. Protein concentrations were determined by BCA (Pierce).

A customised ProcartaPlex 17-plex immunoassay (BAFF, IFNα, IFNβ, IFNγ, IL-1α, IL-1β, IL-10, IL-2, IL-27, IL-33, IL-33R (ST2), IL-4, IL-6, M-CSF, MIP-1α (CCL3), MIP-1β (CCL4), TNFα) was performed on a Bio-Plex 200 (Bio-Rad) platform with Bio-Plex Manager 6.1 software (Bio-Rad) according to the manufacturer’s protocol with the following modifications: universal assay buffer was omitted, and cell lysis buffer was used in place of the reading buffer. From similar starting concentrations, EC and DCN samples were diluted 2X in buffer to obtain sufficient input for analysis and analysed separately from cortex and cerebellum samples to avoid potential biases in quantification. An 8-plex ProcartaPlex immunoassay (BAFF, IL-27, IL-33, IL-6, M-CSF, CCL3, CCL4 and TNFα) was performed on EC and DCN WT and *App* KI samples following PLX5622 treatment according to the manufacturer’s protocol. Technical duplicates were used for both runs.

Analyte concentrations (pg/ml) were calculated from median fluorescence intensities (MFIs) and adjusted for dilution using Milliplex Analyst software (VigeneTech), then normalised by input protein concentrations (pg/mg). Two-way (region * genotype) or three-way (region * genotype * treatment) ANOVAs were conducted on log_2_-transformed concentrations for each protein with Tukey’s HSD post hoc tests.  Full statistical test results are presented in Additional File 2. Heatmaps display z-scores of log_2_-transformed values with Ward D2 clustering.

### RNA analyses of BV2 cells treated with EC or DCN homogenates

EC and DCN tissues were extracted as described for immunoassays, except that 100 µl cold DPBS was used in place of cell lysis buffer. BV2 microglial cell lines were maintained in DMEM (Nacalai Tesque) containing 10% FBS (Gibco) and 1% Pen-Strep (Gibco). One day before treatment, 5 × 10^4^ cells were seeded per well in a 24-well plate. Cells were treated with either 50 μg EC or DCN protein homogenates prepared from *N* = 8 WT male mice aged 12–13 months, an equivalent volume of DPBS as negative control, or 10 ng/ml of lipopolysaccharide (Sigma-Aldrich) as a positive control for induction of inflammatory gene expression. Cells were then incubated for 4 h at 37 °C and washed with ice-cold DPBS, before total RNA was harvested using Arcturus PicoPure RNA Isolation Kit (Applied Biosystems).

For RT-PCR, 1–2 μg of RNA was converted to cDNA using the RevertAid First Strand cDNA Synthesis Kit (Thermo Fisher) with oligo (dT)18 primers. qPCR was carried out using PowerUp SYBR Green Master Mix (Applied Biosystems) on QuantStudio 6 Flex Real-Time PCR System (Applied Biosystems) for all targets. To calculate the ΔCt value, the mean Ct value of technical triplicates for each target gene was normalised against the mean Ct value for the reference gene, *Hprt1*. The ΔCt value was then normalised against the DPBS control to obtain the ΔΔCt value, and the log_2_-fold changes of ΔΔCT values were presented.

### Primers used for qPCR validation

**Table:** 

Gene	Forward primer (5ʹ-3ʹ)	Reverse primer (5ʹ-3ʹ)
*Hprt1*	TGTTGTTGGATATGCCCTTG	GGCCACAGGACTAGAACACC
*Tnf-α*	CAGGCGGTGCCTATGTCTC	CGATCACCCCGAAGTTCAGTAG
*Il-6*	TAGTCCTTCCTACCCCAATTTCC	TTGGTCCTTAGCCACTCCTTC
*Il-1b*	GCCCATCCTCTGTGACTCAT	AGGCCACAGGTATTTTGTCG
*Csf1*	CATCCAGGCAGAGACTGACA	CTTGCTGATCCTCCTTCCAG
*Ifi204*	CAGGGAAAATGGAAGTGGTG	CAGAGAGGTTCTCCCGACTG
*Tlr1*	GTCAAAGCTTGGAAAGAATCTGAAG	AATGAAGGAATTCCACGTTGTTTC
*H2-K1*	GAGCCCCGGTACATGGAA	CAGGTAGGCCCTGAGTCT

For bulk RNAseq, polyA-enriched libraries were prepared and sequenced on an Illumina NovaSeq 6000 platform by NovogeneAIT to obtain 20 M 150-bp paired-end reads per sample. All samples had RNA integrity number (RIN) > 9. Pre-processing of RNAseq data were performed on the Gekko high-performance computing cluster at Nanyang Technological University. Illumina adapters were trimmed and reads with quality score < 15 or length < 30 bp were removed using Trimmomatic v0.39 [121]. Alignment and quantification of paired‐end reads to the Genome Reference Consortium mouse genome primary assembly GRCm39 (annotation vM28) were performed using STAR v2.7.1a [122] and HTseq. v0.11.2 [123]. Downstream analyses were conducted in R 4.2.1 [124]. First, the dataset was filtered to genes with minimum 1 log_2_ count per million (CPM) in at least 3 samples (11,404 genes). Differential expression analyses were conducted on TMM-normalised counts using the quasi-likelihood method with robust dispersion estimation in edgeR [125–127] comparing DCN, EC and LPS conditions to DPBS control, and DCN and EC conditions. Thresholds for differentially expressed genes were false discovery rate (FDR) < 10% and LFC >  ± 1. Full differential expression test results are presented in Additional File 3. A heatmap was plotted showing z-scores for expression in each sample (log_2_ CPM + 1). Hierarchical clustering of genes was performed using Pearson’s correlation with the Ward D2 algorithm. GO analyses were conducted for each ontology (MF, CC, BP) using Fisher’s exact test with the weight01 algorithm in topGO [119, 120].

### Microglia depletion

Chow containing 1200 ppm PLX5622 CSF1R inhibitor (MedChemExpress) was prepared using Open Standard diet (D11112201; Research Diets Inc., USA). Male mice were group-housed in an SPF environment and fed for 60 days with PLX5622 or control diet beginning at age 4 months. Chow was stored in a vacuum-sealed bag at 4 °C and replaced every 3 days. Histology and immunofluorescence staining were performed as described above using IBA1 and 6E10 antibodies and FSB dye. Images were captured at 20X magnification using Zeiss LSM 810 Confocal Microscope System with the same parameters for all sections. Images were analysed using Fiji (NIH). First, the outline of ROIs was drawn, excluding areas of high background or tissue deformation. A Gaussian blur filter was applied, followed by rolling ball background correction. Minimum and maximum thresholds were set for each region and channel for conversion to a binary image, followed by identification and measurement of particles greater than 10 µm (all channels) and 100 µm (FSB and 6E10). Data were compiled and analysed in R. Mean parameter values were calculated from replicate sections (*n* = 3) for each subject (*N* = 9) and used for plots and Student’s *t*-tests. Full statistical test results are presented in Additional File 2. Brightness and contrast were enhanced for presented images.

### Supplementary Information

**Additional file 1: Figure S1. **Schematic of EC and DCN dissection. **Figure S2.** Workflow for snRNAseq. **Figure S3.** SnRNAseq breakdown of number of cells per cell type and condition. **Figure S4.** Proportion of cells of each type isolated for each condition. **Figure S5.** Pseudo-bulk heatmaps showing average expression of genes differentially expressed between APP and WT genotypes in the EC or DCN for each cell type. Heatmaps show expression in each condition in all major cell-types with z-scores calculated for each gene (row). Conditions and cell-types are indicated by colour-coded bar above each heatmap. DEGs in the EC and DCN respectively are indicated by colour-coded bars to the left of each heatmap. Hierarchical clustering was applied to genes in each heatmap. (A) Astrocyte (B) Neurons (C) Microglia (D) Oligodendrocytes (E) OPCs (F) Vascular cells. **Figure S6.** (A) UMAP of module Mg.G4 as a percentage of the maximum expression of genes found in the module. (B) Expression UMAPs for selected top marker genes from module Mg.G4 showing percentage of maximum expression. (C) Gene Ontology analysis of biological processes enriched in module Mg.G4 compared to all genes detected in microglia. **Figure S7.** (A) Expression pattern of *Hexb* (left panels) in the cortex and the cerebellum. On the right is an illustration of *Hexb* aggregates sized between 10 to 100 µm^2^ marked by grey dots while aggregates sized larger than 100 µm^2^ marked in red. Dashed lines in the cortical region indicates the EC region. Dashed lines in the cerebellum demarcates the DCN region while the grey shaded area denotes the white matter (WM) region. Scale bar indicates 1000 µm. (B) Quantification of the number of *Hexb* population in the EC, DCN and WM of WT and APP tissue (N=3 subjects, n=6-7 sections/subject). Graph indicates mean ± SEM. (C) RNA *in situ* hybridisation of *Oasl2* and *Ifi204* (green) in the DCN and WM of APP tissue. Closed circle denotes colocalisation with *Hexb* (red) while broken circle denotes absence of *Oasl2* or *Ifi204* in *Hexb*-positive cells. Scale bar represents 10 µm. (D-E) Quantification of *Oasl2*-expressing microglia (D) and *Ifi204*-expressing microglia (E) in the DCN and WM (N=3 subjects, n=3 sections/subject). Graphs indicate mean ± SEM. Student’s t-tests were performed on within region (*) and within-genotype differences in DCN or WM as compared to EC (†). Two-way ANOVAs were performed on within-subject means to test for main effects of region and genotype and interaction effects, followed by Tukey’s HSD post hoc tests (¤). * p<0.05, ** p<0.01, *** p<0.001, **** p<0.0001. **Figure S8.** (A-D) UMAPs showing (i) cell subclusters, (ii) distribution of cells across conditions and (iii) aggregated expression of genes in each module, and (iv) box plots of log_2_ gene expression value distributions for each gene module for (A) Astrocytes, (B) Oligodendrocytes, (C) OPCs and (D) Vascular cells. **Figure S9.** Dot plots showing expression of select DEGs associated with innate immune responses, cytokine production and NFKB signalling in modules Vasc.2, Vasc.4, and Olig.3 across conditions. Dot size indicates the percentage of cells in each condition expressing each gene and dot colour indicates mean log_2_ expression. Darker dots represent higher expression levels. **Figure S10.** Cytokine protein expression measured by multiplex array in WT and *App* KI tissues. Bar plots show mean and SEM for each condition with dots for individual sample measurements. Region and genotype are indicated by bar colour and shade. Significant post hoc t-test results are indicated as follows: * p<0.05, ** p<0.01, *** p<0.001, **** p<0.0001. (A) EC & DCN. (B) Cortex (CTX) and cerebellum (CBM). **Figure S11.** STRING analysis of DEGs that overlap in DCN and LPS conditions compared to controls in BV2-treated cells. Genes associated with GO label “immune response”, “response to cytokines” and “inflammatory response” are represented as colour coded circles. (Gene interactions shown under medium confidence with no disconnected nodes.). **Figure S12.** Log_2_ fold change in expression of marker genes associated with inflammation in BV2 microglia cultures stimulated with DCN homogenates compared to EC homogenates from the same subject, measured by qPCR (N=8). P-values from paired t-tests are written above each bar. **Figure S13.** Cytokine protein expression measured by multiplex array following microglia depletion by CSF1R inhibition. WT and *App* KI mice were fed with PLX5622 or control chow for 2 months, followed by isolation of the EC and DCN. Bar plots show mean and SEM for each condition with dots for individual sample measurements. Region and genotype are indicated by bar colour, while treatment is indicated by diagonal stripes. Protein concentrations are normalised by total protein in each sample. **Figure S14.** Quantification of IBA1^+^ microglia coverage in the EC and DCN of WT mice fed with PLX5622 and control chow, showing robust depletion in both regions (N=4 subjects, n=2 sections/subject). * p<0.05, *** p<0.001. **Figure S15.** Quantification of 6E10-labeled amyloid plaques in the EC (A) and DCN (B) for the number, total area and average size of two different plaque size thresholds (>10 µm^2^ and >100 µm^2^; N=9 subjects, n=3 sections/subject). **Figure S16.** Expression of *App *and select APP-processing genes in snRNAseq data. (A) UMAPs displaying log_2_ expression of *App *across all cells in each condition. (B) Violin plots of *App* expression in each major cell type and condition. (C) Heatmap of pseudo-bulk average gene expression of *App* and select APP-processing genes in each major cell type in each condition. Colour scale indicates z-scores for each gene across conditions.

**Additional file 2:** Statistical tables for Figures 1, 3, 6, 7, S7, S14 and S15.

**Additional file 3:** Supplementary Data for snRNAseq and RNAseq experiments.

## Data Availability

Sequencing datasets can be downloaded from the NCBI GEO repository, SuperSeries GSE223076. The accession numbers are GSE214244 for the snRNAseq data and GSE223018 for the BV2 RNAseq data. Where reasonable, all materials are available from commercial suppliers or by request.
