# Supplementary material for: Cytokine enrichment in deep cerebellar nuclei is contributed by multiple glial populations and linked to reduced amyloid plaque pathology
Source: J Neuroinflammation. 2023 Nov 17;20:269. doi: 10.1186/s12974-023-02913-8 (PMC10656954; doi:10.1186/s12974-023-02913-8)

Supplementary Figure

FIGURE S1

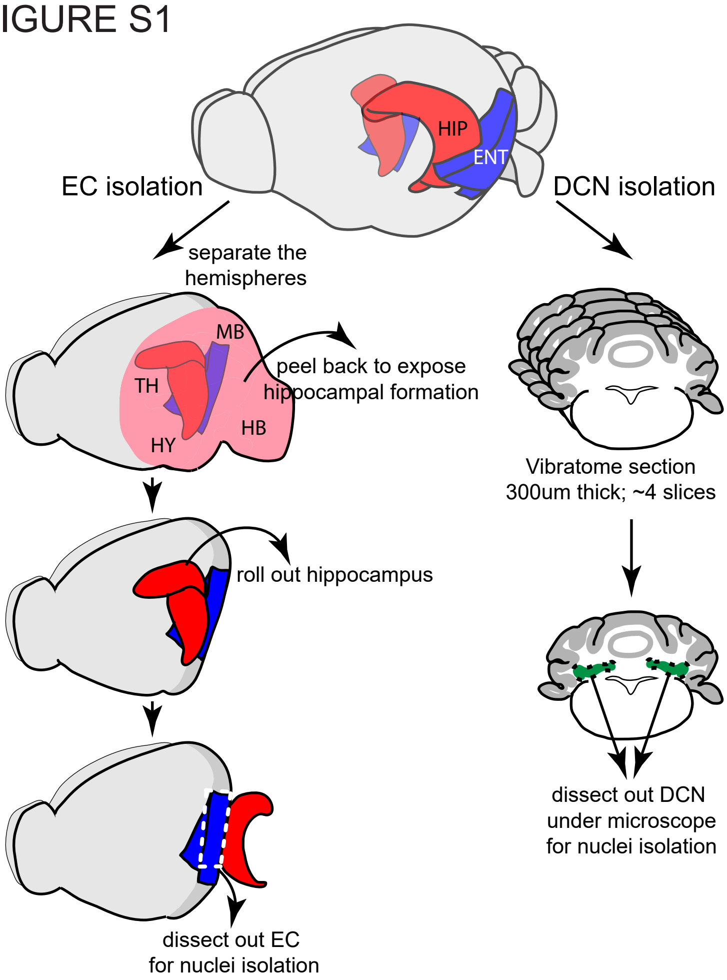

FIGURE S2

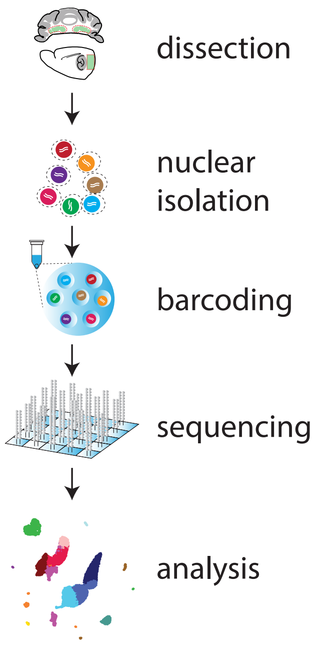

FIGURE S3

Cell-type	EC.WT	EC.APP	DCN.WT	DCN.APP
Astrocyte 1	1606	3075	634	954
Astrocyte 2	1326	1634	1081	1825
Bergmann glia	0	0	170	57
COP	61	78	27	42
Cajal Retzius cells	46	23	0	0
Vascular endothelial	276	571	231	571
Ependymal	6	13	25	107
Excitatory neuron	8254	10273	10760	8761
Interneuron	1393	1695	484	895
Leptomeningeal	49	105	66	84
Microglia	1092	2119	287	669
Oligodendrocyte 2	876	1413	2643	4381
Oligodendrocyte 1	1095	1631	2305	4149
OPC	645	1064	185	413
Pericyte	192	385	134	226
Peripheral immune	21	87	12	50

FIGURE S4

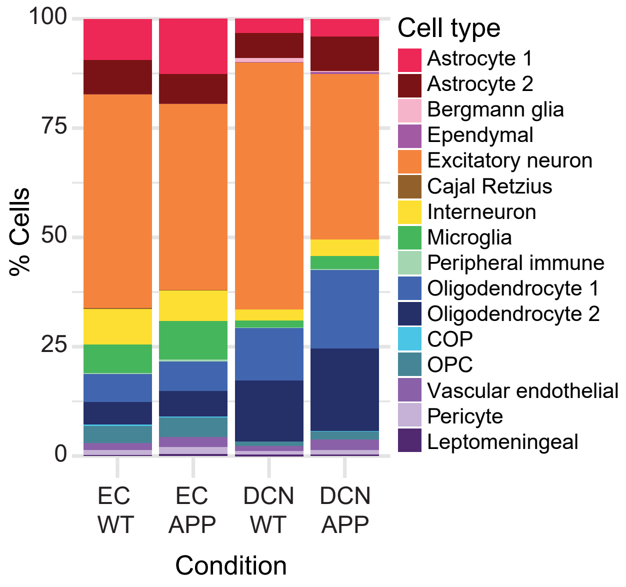

Supplementary Figure

FIGURE S5

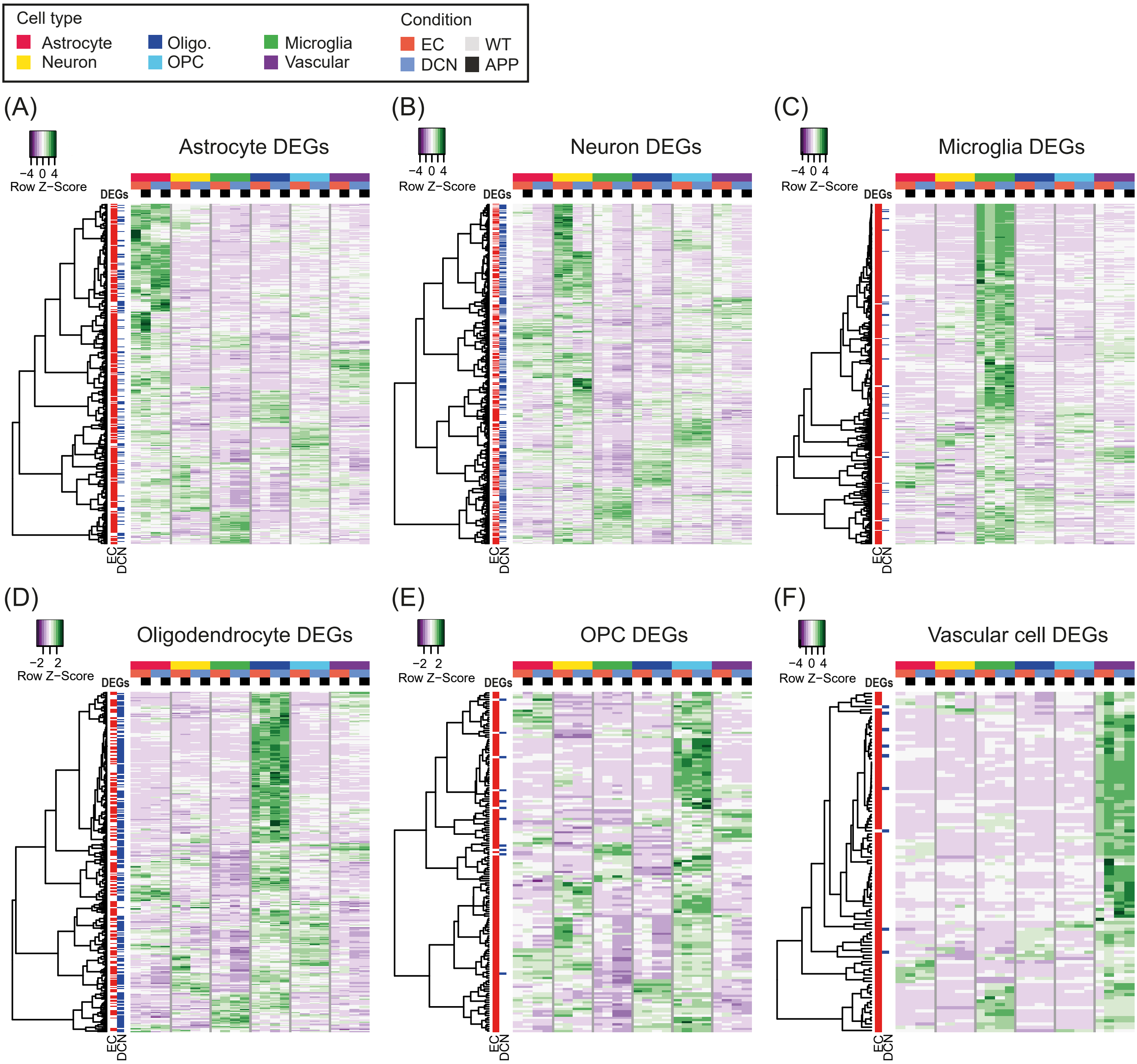

Supplementary Figure

FIGURE S6

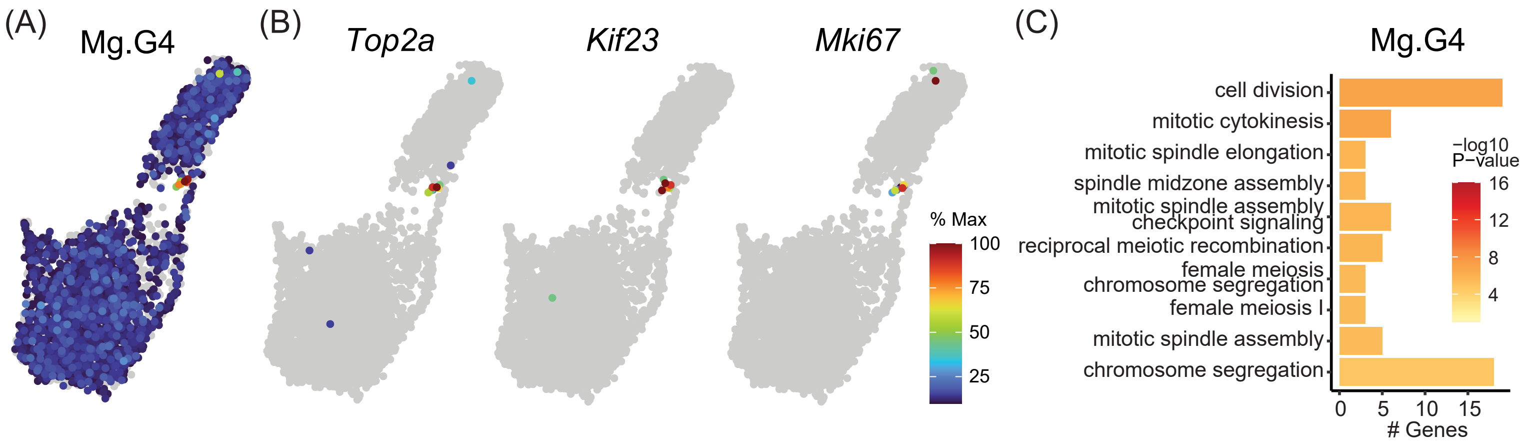

FIGURE S7

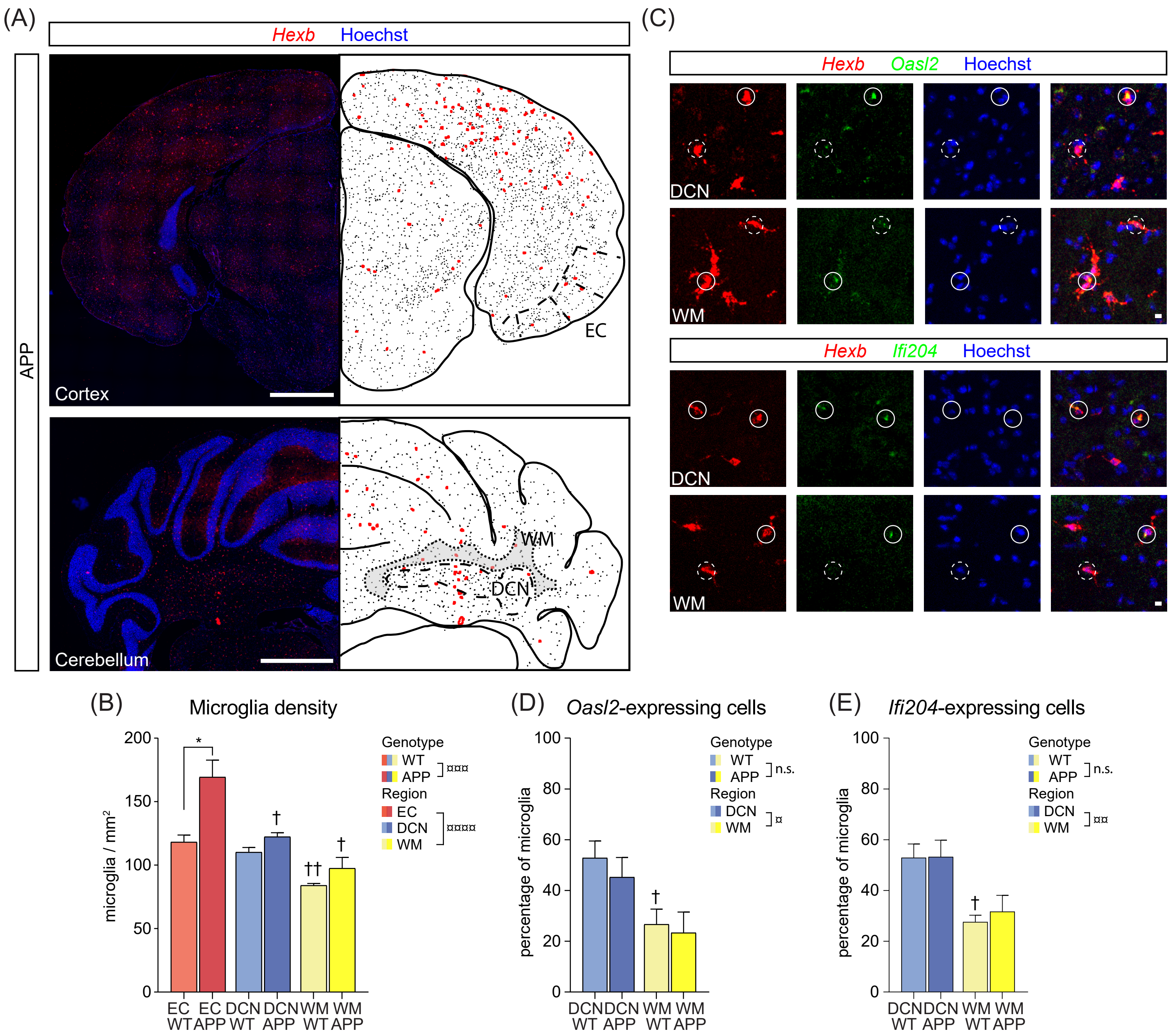

Supplementary Figure

FIGURE S8

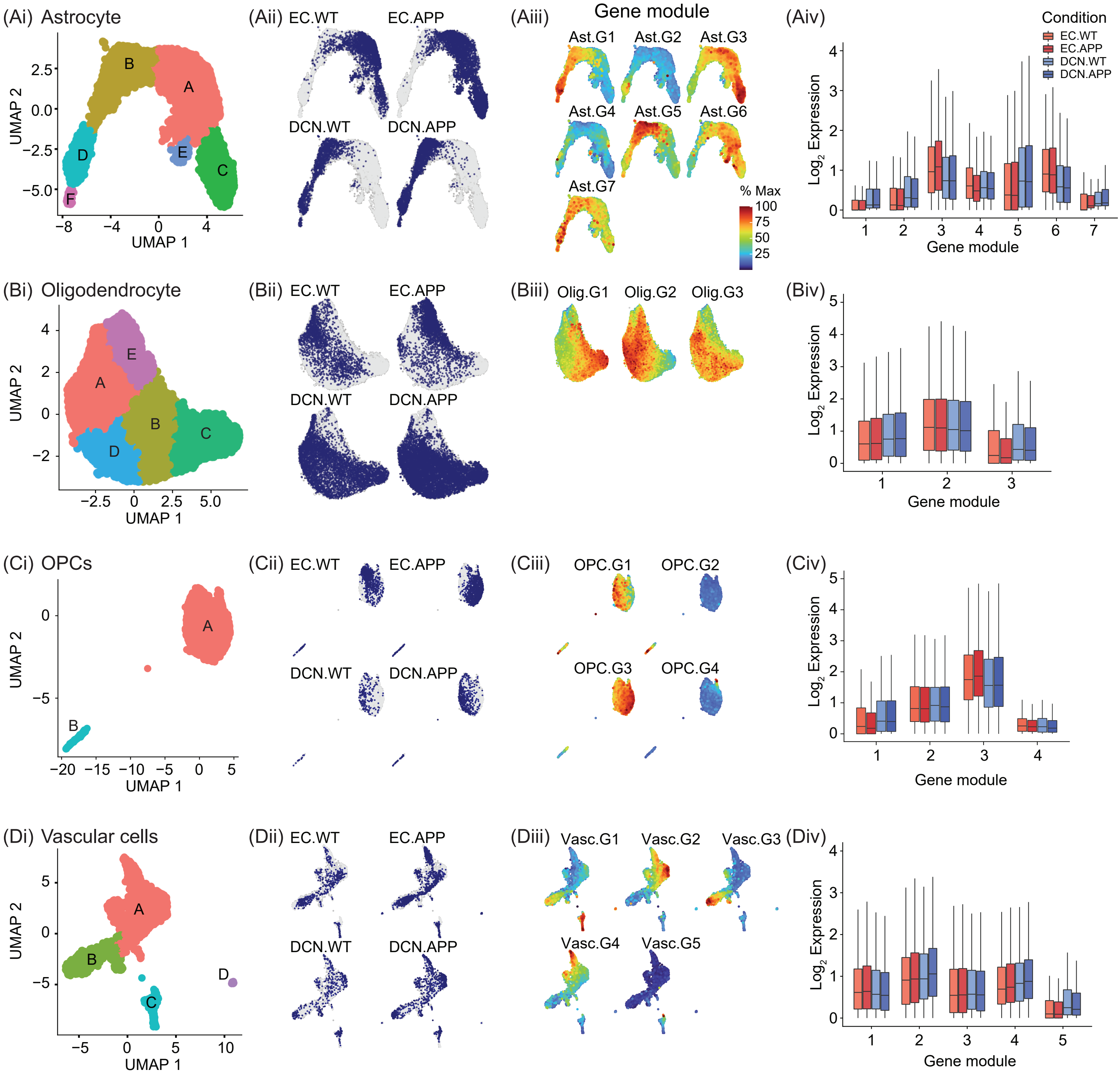

FIGURE S9

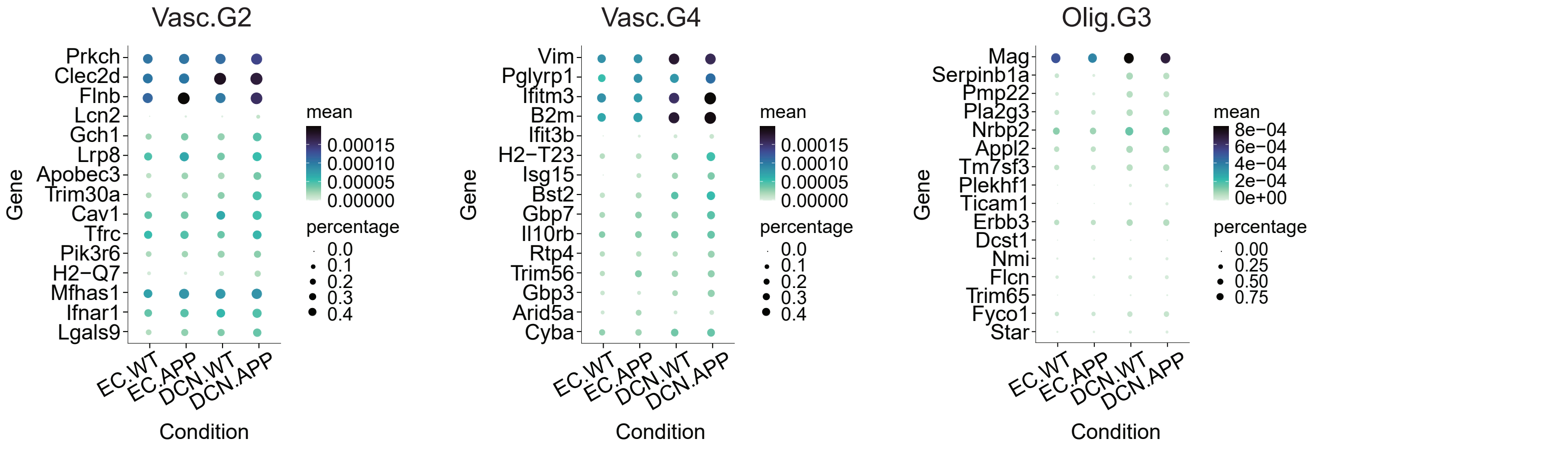

Supplementary Figure

FIGURE S10

(A)

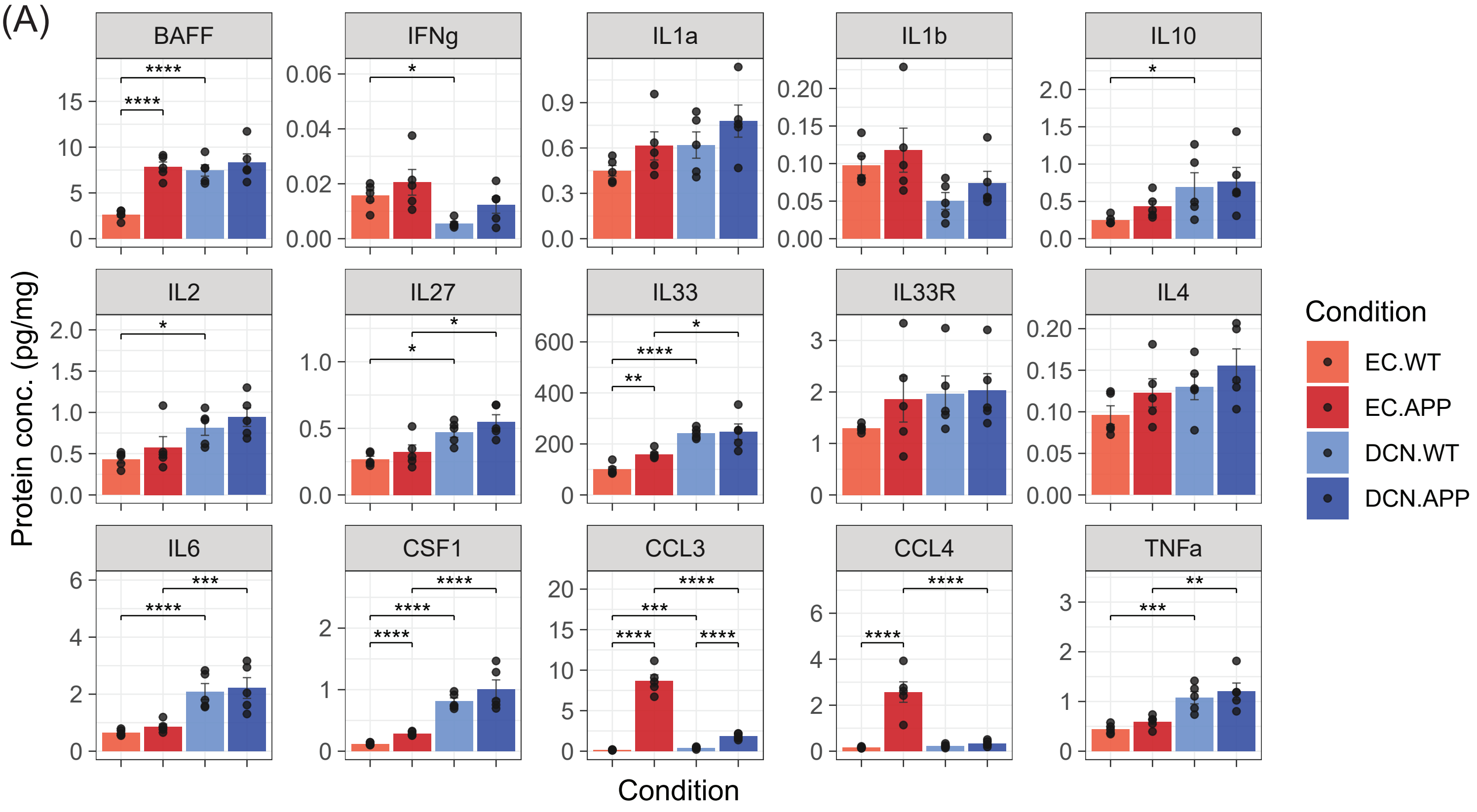

(B)

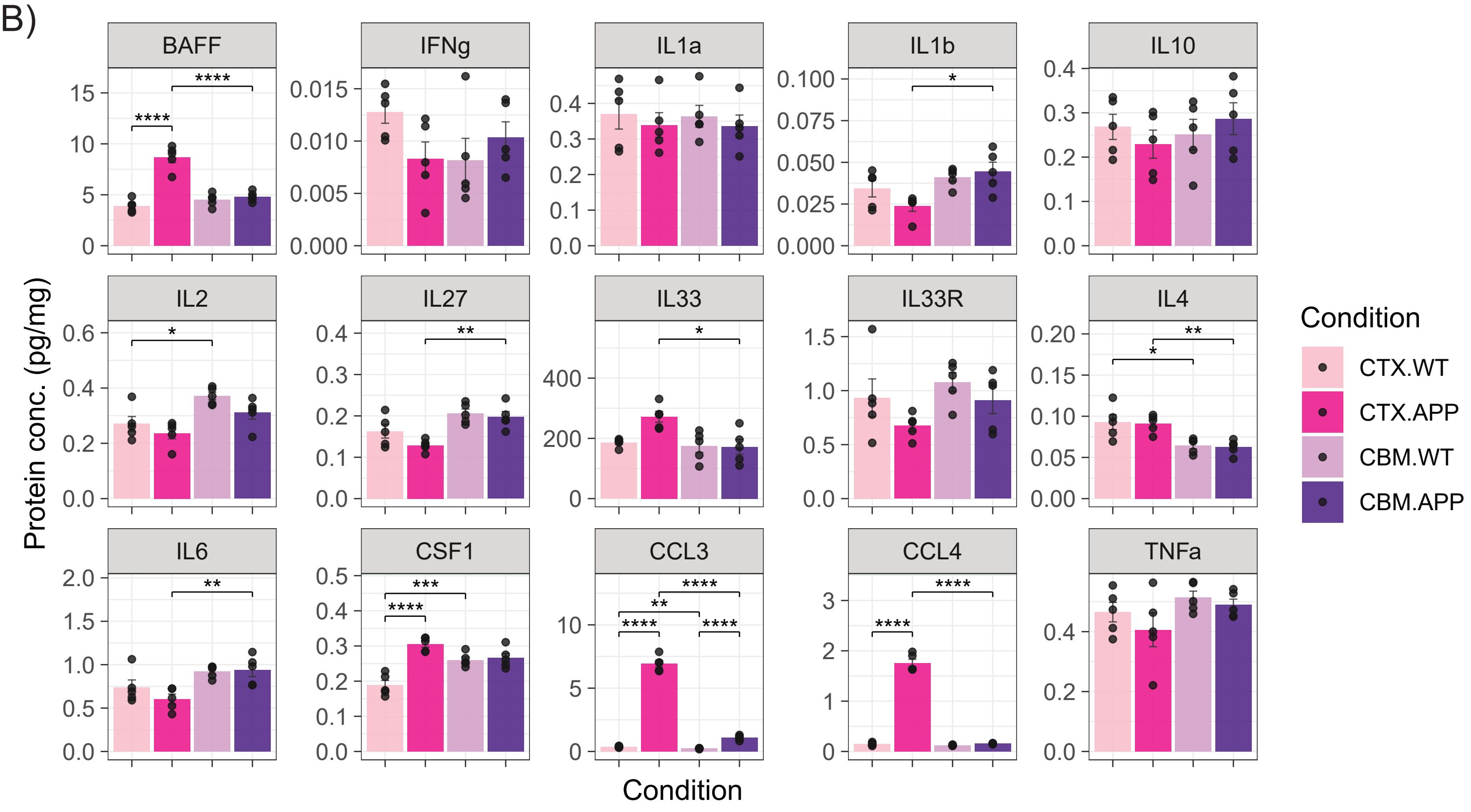

Supplementary Figure

FIGURE S11

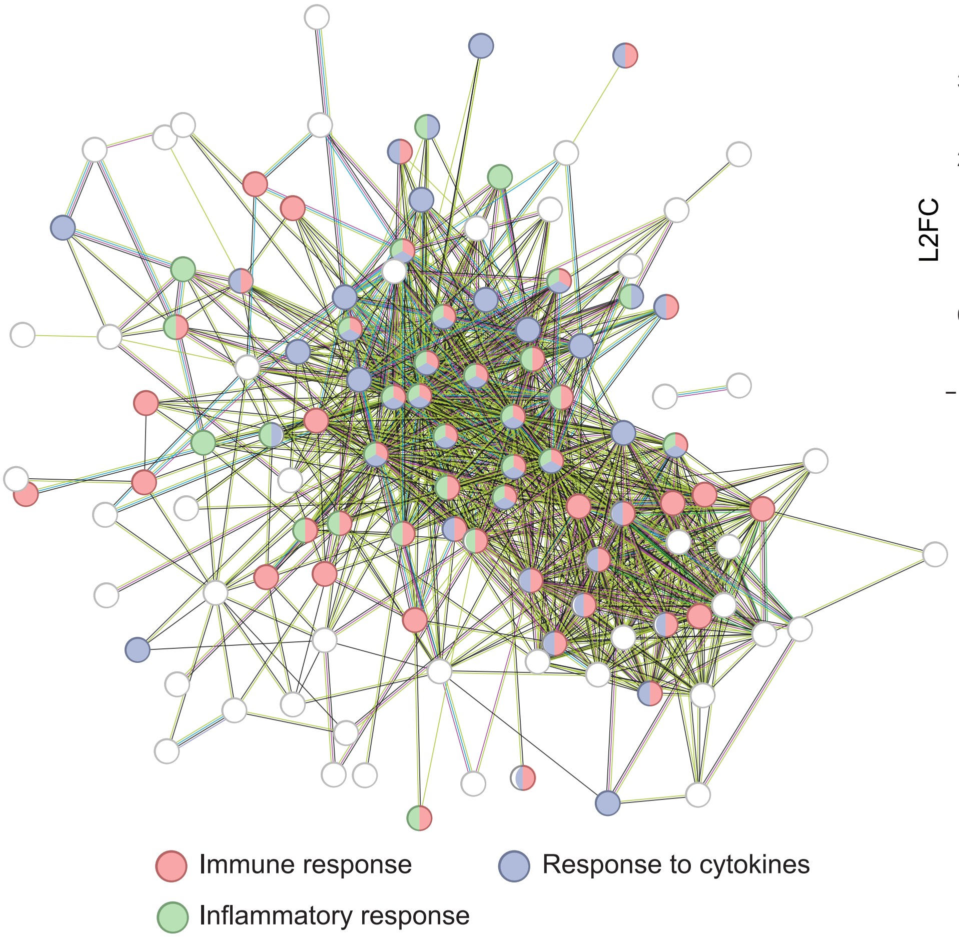

FIGURE S12

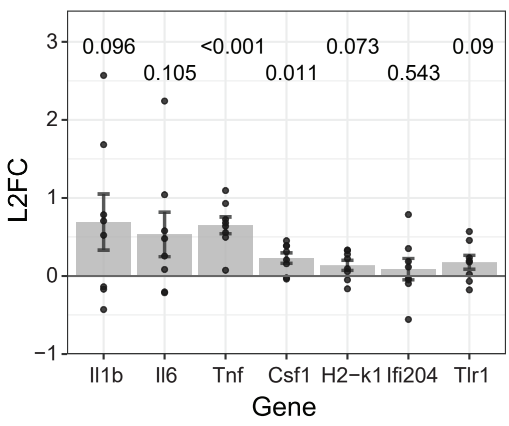

FIGURE S13

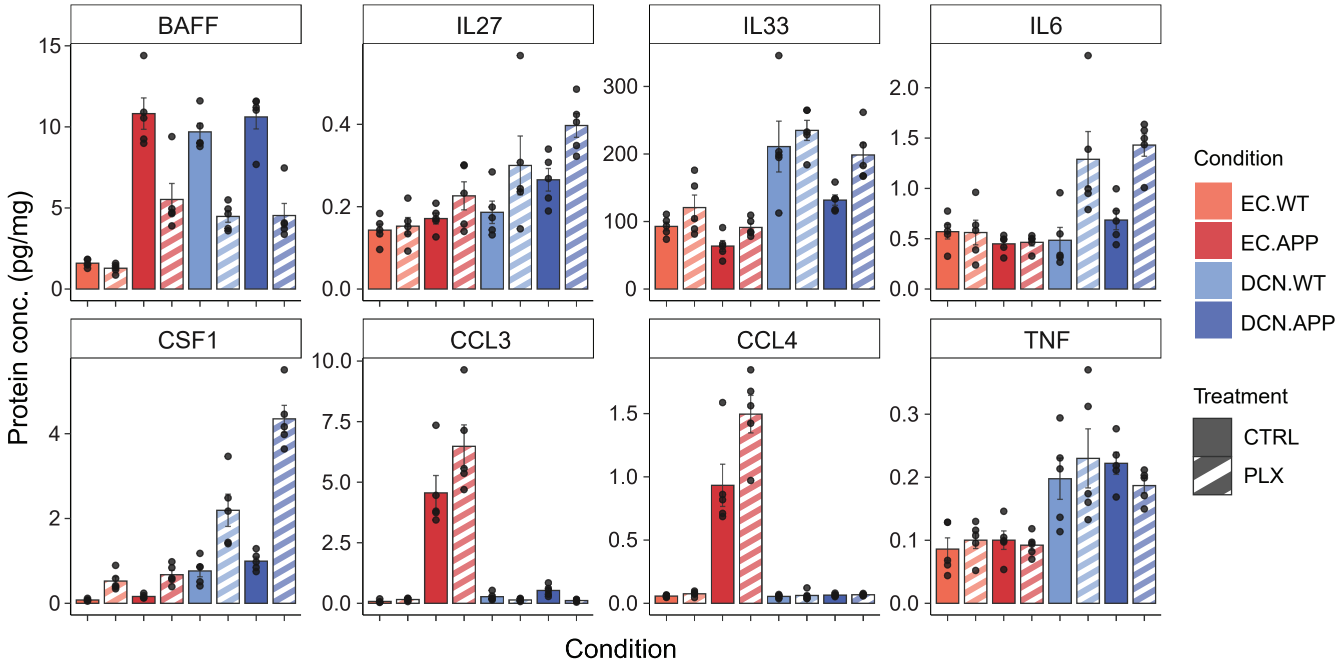

Supplementary Figure

FIGURE S14

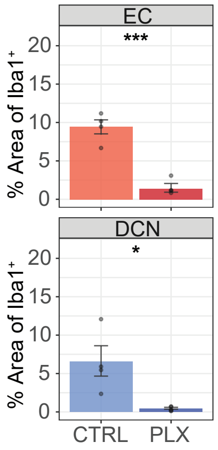

FIGURE S15

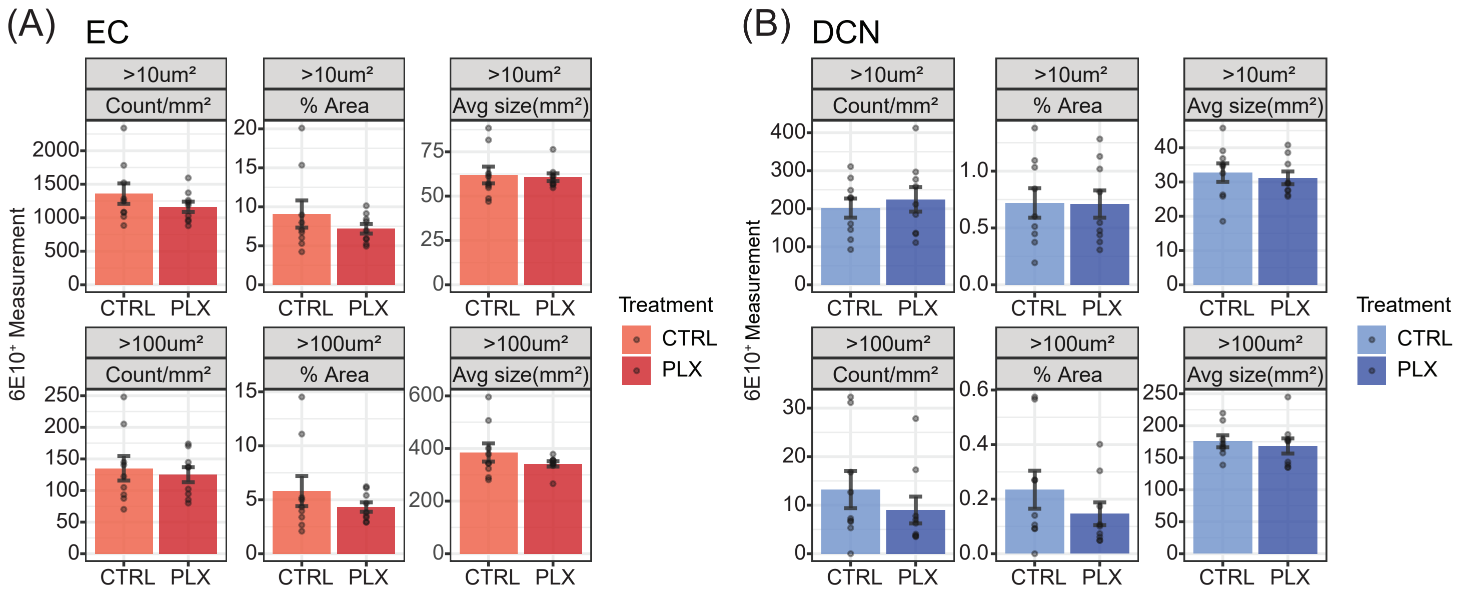

Supplementary Figure

FIGURE S16

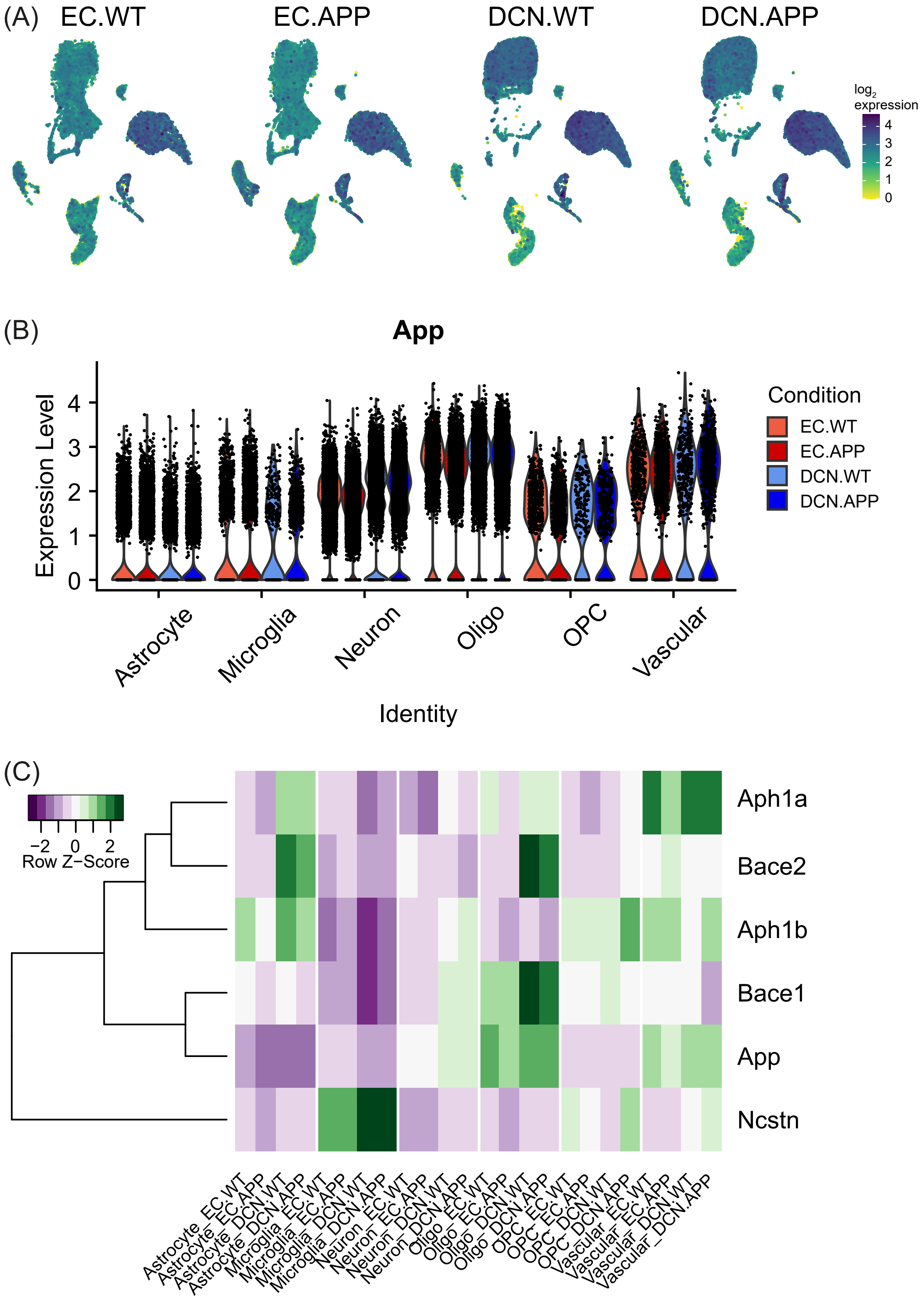

Supplement: Supplementary file 1 — Additional file 1: Figure S1. Schematic of EC and DCN dissection. Figure S2. Workflow for snRNAseq. Figure S3. SnRNAseq breakdown of number of cells per cell type and condition. Figure S4. Proportion of cells of each type isolated for each condition. Figure S5. Pseudo-bulk heatmaps showing average expression of genes differentially expressed between APP and WT genotypes in the EC or DCN for each cell type. Heatmaps show expression in each condition in all major cell-types with z-scores calculated for each gene (row). Conditions and cell-types are indicated by colour-coded bar above each heatmap. DEGs in the EC and DCN respectively are indicated by colour-coded bars to the left of each heatmap. Hierarchical clustering was applied to genes in each heatmap. (A) Astrocyte (B) Neurons (C) Microglia (D) Oligodendrocytes (E) OPCs (F) Vascular cells. Figure S6. (A) UMAP of module Mg.G4 as a percentage of the maximum expression of genes found in the module. (B) Expression UMAPs for selected top marker genes from module Mg.G4 showing percentage of maximum expression. (C) Gene Ontology analysis of biological processes enriched in module Mg.G4 compared to all genes detected in microglia. Figure S7. (A) Expression pattern of Hexb (left panels) in the cortex and the cerebellum. On the right is an illustration of Hexb aggregates sized between 10 to 100 µm2 marked by grey dots while aggregates sized larger than 100 µm2 marked in red. Dashed lines in the cortical region indicates the EC region. Dashed lines in the cerebellum demarcates the DCN region while the grey shaded area denotes the white matter (WM) region. Scale bar indicates 1000 µm. (B) Quantification of the number of Hexb population in the EC, DCN and WM of WT and APP tissue (N=3 subjects, n=6-7 sections/subject). Graph indicates mean ± SEM. (C) RNA in situ hybridisation of Oasl2 and Ifi204 (green) in the DCN and WM of APP tissue. Closed circle denotes colocalisation with Hexb (red) while broken circle denotes absence of [file 12974_2023_2913_MOESM1_ESM.pdf]
